# High-Entropy Layered Hydroxides: Pioneering Synthesis, Mechanistic Insights, and Multifunctional Applications in Sustainable Energy and Biomedicine

**DOI:** 10.1007/s40820-025-02023-5

**Published:** 2026-01-07

**Authors:** Zhengqian Jin, Zhenjiang Cao, Li Jin, Shujiang Ding, Kai Xi

**Affiliations:** School of Chemistry, Engineering Research Center of Energy Storage Materials and Devices, Ministry of Education, National Innovation Platform (Center) for Industry-Education Integration of Energy Storage Technology, Engineering Research Center of Energy Storage Material and Chemistry, Universities of Shaanxi Province, Xi’an Jiaotong University, Xi’an, 710049 People’s Republic of China

**Keywords:** High-entropy, Layered hydroxides, Energy storage, Energy conversion, Biomedical applications

## Abstract

High-entropy layered hydroxides (HELHs), an emerging frontier in entropy-stabilized materials derived from layered double hydroxides (LDHs), have captivated attention with their unparalleled tunability, thermodynamic stability, and electrochemical performance. The integration of the high-entropy concept into LDHs empowers HELHs to surmount the constraints of conventional materials through compositional diversity, structurally disordered configurations, and synergistic multi-element interactions. This review systematically embarks on their synthesis methodologies, functional mechanisms, and applications in energy conversion/storage and biomedicine. Advanced synthesis strategies, such as plasma-assisted hydrothermal methods, facilitate precise control over HELH architectures while supporting scalable production. HELHs demonstrate superior electrochemical performance in critical reactions, including oxygen evolution reaction, water oxidation, hydrogen evolution, and glucose electrooxidation. Future directions encompass integrating in situ characterization with simulations, leveraging machine learning for composition screening, and expanding HELHs application through interdisciplinary collaborations. This work establishes a comprehensive roadmap for advancing HELHs as next-generation multifunctional platforms for sustainable energy and biomedical technologies.

## Highlights

- Synthesis Methodologies: We systematically investigate co-precipitation, framework-guided, and plasma-assisted hydrothermal methods for the synthesis of high-entropy layered hydroxides (HELHs), achieving precise control over the porosity, surface chemistry, and interfacial properties of ultrathin nanosheets through atomic-level mixing and defect engineering.
- Functional Mechanisms: HELHs possess compositional disorder, synergistic interactions among multiple components, lattice distortion-induced active sites, and inherent structural stability, collectively contributing to their superior electrochemical performance.
- Multifunctional Applications: HELHs excel as oxygen/hydrogen evolution reactions electrocatalysts for energy devices and enable photocatalytic reactive oxygen species generation for cancer treatment, underscoring their dual potential in sustainable energy conversion and biomedical therapeutics.

## Graphical abstract

**Figure:**
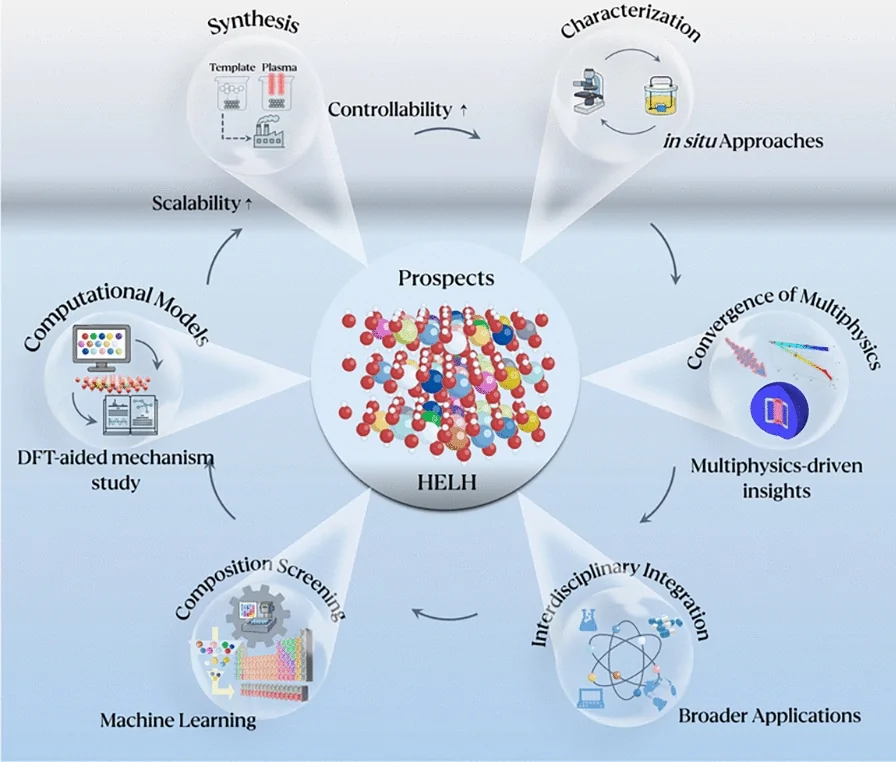

## Introduction

The burgeoning advancement of emerging energy and biomedical technologies has created unprecedented demand for novel materials featuring innovative synthesis strategies and in-depth mechanistic understanding. This demand spans sustainable energy applications through next-generation batteries [1–4], supercapacitors [5–8], and electrochemical conversion systems [9, 10], as well as frontier biomedical domains. However, the development of these technologies is impeded by significant challenges, such as the imperative to enhance energy density [11], extend cycle life [12], and ensure environmental sustainability [13]. From a materials science perspective, a promising strategy has emerged to address these limitations through entropy engineering, which overcomes the inherent constraints of conventional materials. This approach has led to the development of a new class of materials known as high-entropy materials (HEMs). HEMs possess distinctive characteristics, such as multi-component compositions, structurally disordered configurations, and multiphase architectures that enable precise tailoring to meet specific application requirements [14, 15]. A material is considered to exhibit high-entropy characteristics when its configurational entropy (ΔS_conf_) is equal to or exceeds 1.5R, where R denotes the gas constant [14, 15]. This value can be calculated for an ideal solid solution using the Boltzmann equation:

1 $$\begin{array}{*{20}c} {{\Delta S}_{{{\mathrm{conf}}}} = - R\mathop \sum \limits_{i = 1}^{n} x_{i} \ln x_{i} } \\ \end{array}$$

where *n* is the number of principal elements and *x* is the molar fraction of the i-th element. To maximize this configurational entropy and promote the formation of a single-phase solid solution, equimolar ratios (*x*_*i*_ = 1/*n*) are commonly employed. In recent years, the concept of high entropy has been incorporated into various material systems, including the pioneering high-entropy alloys (HEAs) [16–19], high-entropy oxides (HEOs) [20], high-entropy ceramics (HECs) [21], high-entropy zeolitic imidazolate frameworks (HE-ZIFs) [22], high-entropy Prussian blue analogues (HE-PBAs) [23], high-entropy oxyfluorides (HEOFs) [24], high-entropy selenides (HESes) [25], high-entropy sulfides (HESs) [26, 27], high-entropy MXenes (HEMXs) [28, 29], high-entropy van der Waals materials [30], and others. The pursuit of HEMs has catalyzed transformative advances in energy storage and conversion technologies. Among these, HEAs and HEOs have demonstrated exceptional performance through their multi-elemental synergy and entropy-stabilized configurations. Building upon this foundation, high-entropy layered hydroxides have emerged as a structurally distinct subclass, inheriting and amplifying the advantages of conventional layered double hydroxides (LDHs) while integrating high-entropy design principles.

LDHs represent as a distinct class of typical two-dimensional materials, distinguished by their layered structure. Comprising high surface areas and reactivity, LDHs feature positively charged metal hydroxide layers that accommodate a variety of intercalated anions and solvent molecules within their interlayer galleries [31, 32]. It is noteworthy that numerous materials with analogous structures, such as montmorillonite [33] and other clay minerals [34], feature negatively charged main metal layers and positively charged ions within their intercalated layers. In contrast, LDHs serve as a rare class of anion exchange materials, wherein their multifunctionality primarily stems from their tunable chemical composition and ion exchange properties. This versatility is exemplified by the tunable oxidation states and transition metal doping, rendering LDHs as pivotal materials for applications in energy storage and catalysis.

Contributing to their customizable properties, LDHs can be engineered into hybrid nanostructures featuring 3D hierarchical porous frameworks and heterointerfaces, which enhance electrical conductivity and stability. These hybrid LDHs are actively investigated as multifunctional nanomaterials for applications in water splitting and supercapacitors [35]. Moreover, the high anion exchange capacity of LDHs has been extensively exploited for chelating pollutants, such as phosphate [36] and arsenate [37, 38] from water systems. LDHs also serve as a versatile platform for developing advanced materials for ion separation and energy technologies. Their unique layered architecture enables integration with diverse functional materials to construct tailored heterostructures. For instance, synergistic interactions at the interfaces of 2D/2D composites, such as those combining LDHs with highly conductive MXenes, can significantly boost electrochemical performance by overcoming the intrinsic limitations of individual components [39]. Similarly, the porous framework of LDHs facilitates defect engineering and integration with 3D materials like zeolitic imidazolate frameworks (ZIFs), a strategy demonstrated to simultaneously improve membrane permeability and selectivity for ion separation [40]. Collectively, these examples underscore the exceptional versatility of LDHs in forming composites with a broad range of materials—from 2D nanosheets to 3D frameworks—to achieve precisely tailored functionalities.

High-entropy layered hydroxides (HELHs) overcome the limitations of traditional LDHs by integrating the high-entropy paradigm into their layered architecture. Their nanostructured lamellar framework features cation-disordered metal hydroxide layers and periodically ordered anionic interlayers, enabling synergistic improvements in ionic conductivity, charge storage dynamics, and lattice stability.

HELHs offer distinct advantages over single-, double-, triple-, or multi-metal atom (SMAs/DMAs/TMAs/MMAs) materials. While multi-metal atom materials, such as sulfides [41], fluorides [42], and MXenes [43], can exhibit high catalytic activity and stability [44], the core advantage of HELHs stems from their unique high-entropy effect. This effect promotes strain dynamics and enhances interlayer anion bonding, thereby conferring superior thermal stability and antipoisoning capabilities. Geometrically, the synthesis of HELHs is relatively straightforward and cost-effective. Methods such as co-precipitation or template-assisted synthesis enable precise control over the stoichiometric ratios of five or more elements, facilitating adaptive structural reconstruction. This structural flexibility enables HELHs to form complex 3D hierarchical structures that harness the synergistic effects of multiple metal components and high-entropy stabilization. From an electronic standpoint, the collaborative interactions among multiple metals in HELHs lead to highly tunable Fermi levels and a rich density of active sites. Such electronic tunability is critical for optimizing catalytic performance by enhancing intermediate adsorption and accelerating reaction kinetics. Ultimately, HELHs transcend the limitations of conventional multi-element composites by leveraging a thermodynamically stable, high-entropy lattice, paving the way for the rational design of advanced functional materials.

In essence, the entropy-driven structural optimization of HELHs manifests in three critical attributes: (1) ultrahigh cycling stability enabled by thermodynamic stabilization mechanisms; (2) atomic-scale lattice distortion generating catalytically active sites; and (3) multivalent cation/anion interactions that accelerate charge transfer kinetics. Such properties position HELHs as frontier materials for extreme-condition applications, from aerospace-grade battery systems to industrial-scale electrocatalytic reactors.

**Fig. 1:**
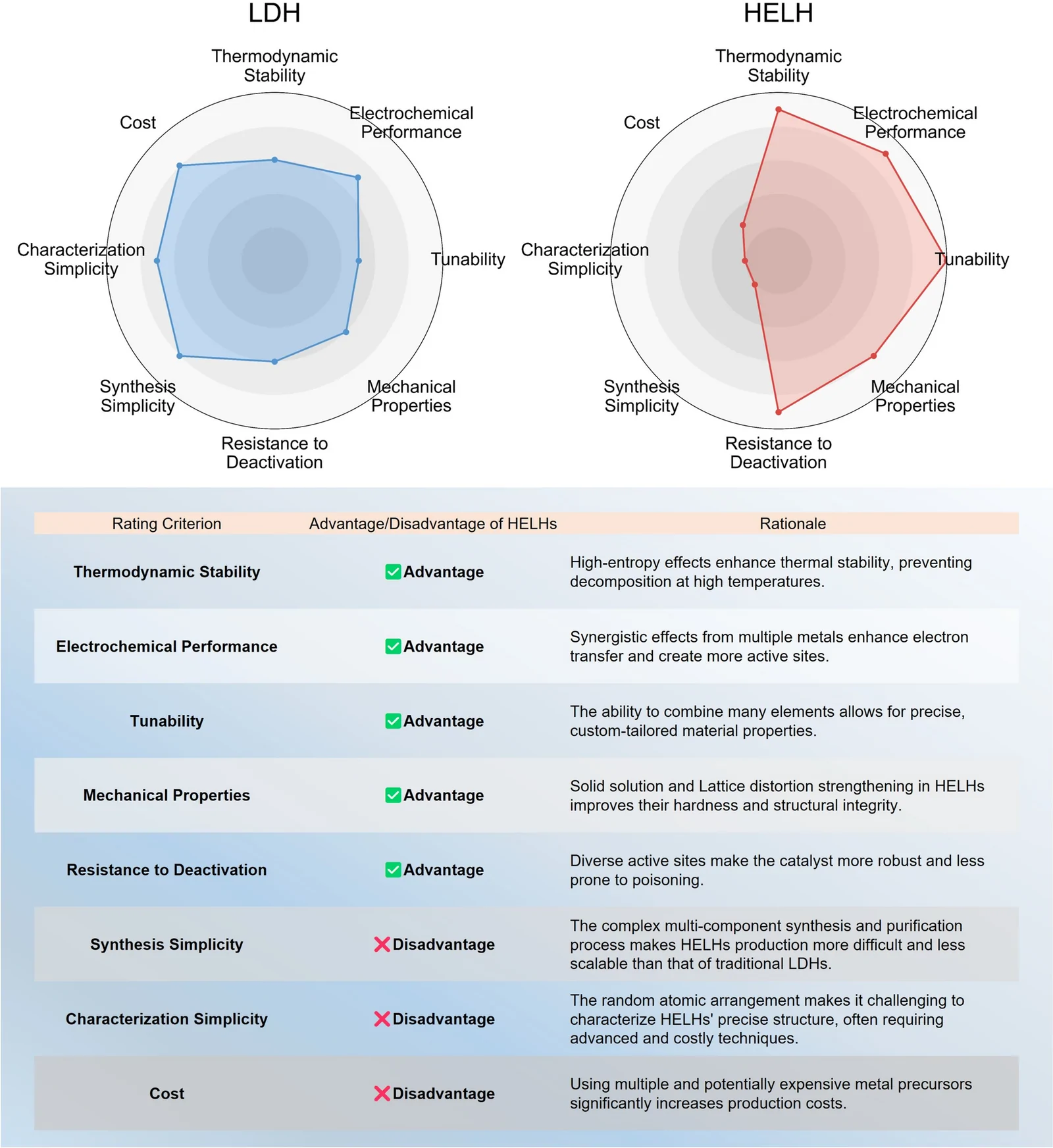
Comparative performance analysis of traditional layered double hydroxides (LDHs) versus high-entropy layered hydroxides (HELHs). The radar charts on top visually depict the performance profiles of both materials across key metrics, with a larger area indicating superior performance. Below the charts, a detailed table outlines the advantages, disadvantages, and rationale for HELHs, providing a comprehensive analysis of their strengths and weaknesses

To elucidate the comparative advantages of HELHs over traditional LDHs, Fig. 1 provides a comprehensive visual and analytical assessment. The radar charts demonstrate that HELHs exhibit a broader performance profile, reflecting their superior overall functionality. This comparative analysis highlights the strengths of HELHs across several key metrics and identifies the principal challenges associated with their development and application. HELHs exhibit superior performance across all material functional categories. The combined effects of high-entropy configuration and multi-metallic synergy confer a distinct advantage in thermodynamic stability and electrochemical performance, enhancing their robustness under harsh conditions and effectiveness in catalysis and energy storage applications. The tunability of HELHs is particularly noteworthy, as the ability to tailor material properties through controlled variation of metal components provides a degree of design flexibility unattainable with traditional LDHs. However, this enhanced performance entails significant trade-offs. HELHs exhibit low ratings in key practical metrics, including synthetic simplicity, ease of characterization, and cost-effectiveness. The complex, multi-component synthesis of HELHs hinders scalable production, while their disordered atomic arrangement presents a significant challenge for structural characterization, frequently necessitating advanced and resource-intensive analytical techniques. The requirement for multiple metal precursors further increases overall production costs. In summary, although HELHs represent a promising advancement in materials science with substantial performance potential, the challenges associated with their synthesis and characterization remain key barriers to industrial-scale implementation.

**Fig. 2:**
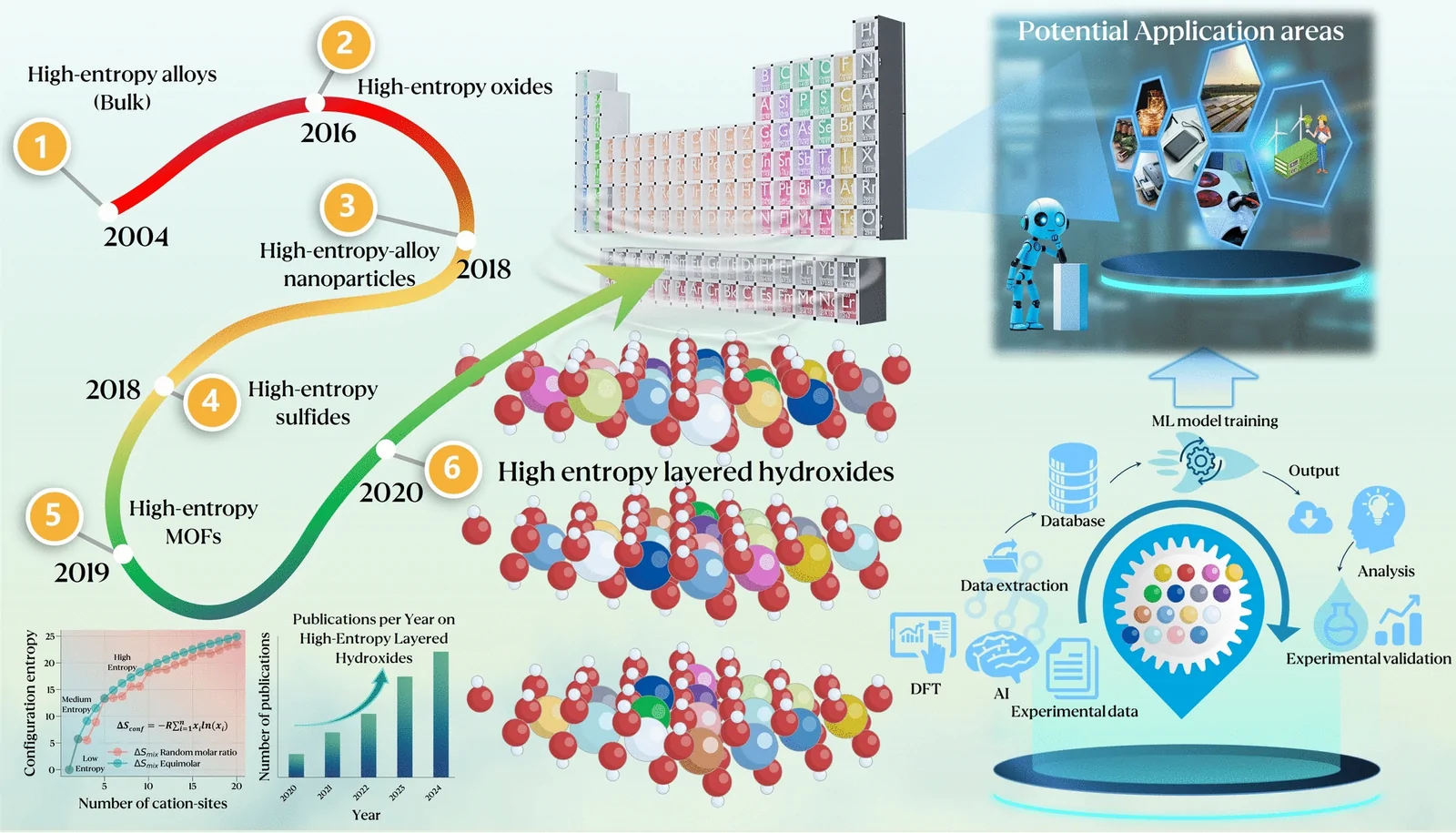
Scheme illustration of evolution of HELHs, with machine learning optimizing element selection to enhance catalytic performance. AI-driven research accelerates their applications in energy conversion and storage

This review provides a rigorous mechanistic analysis of recent breakthroughs in HELHs, systematically deconstructing their synthesis–structure–property relationships across energy and biomedical domains (Fig. 2). The article defines HELHs as entropy-stabilized materials characterized by compositional disorder within layered frameworks, emphasizing their intrinsic advantages—multi-component synergistic effects, lattice distortion-induced active sites, and exceptional thermodynamic/chemical stability. A systematic investigation of synthetic methodologies, including co-precipitation, framework-guided approaches, and plasma-assisted methods, reveals how atomic-level mixing and defect engineering dictate the formation of ultrathin HELH nanosheets, providing insights into tailoring their porosity, surface chemistry, and interfacial properties. In energy-related applications, HELHs demonstrate unparalleled performance as bifunctional electrocatalysts for oxygen/hydrogen evolution reactions (OER/HER). Furthermore, the review highlights the transformative potential of HELHs in biomedicine, where their tunable bandgaps and biocompatibility facilitate photocatalytic reactive oxygen species (ROS) generation for tumor ablation and immune checkpoint modulation. Critical challenges and future directions emphasize four converging priorities: (1) developing scalable methods for synthesizing phase-pure HELHs with controlled anisotropy; (2) implementing operando techniques to monitor cation/anion dynamics during electrochemical cycling; (3) establishing entropy-inclusive density functional theory (DFT) frameworks for predicting metastable phases; and (4) leveraging machine learning algorithms trained on experimental and computational datasets for high-throughput screening of optimal compositions. By integrating materials innovation with AI-driven design strategies, HELHs hold great promise for revolutionizing sustainable energy systems and precision oncology, establishing them as leading candidates among next-generation functional materials.

## Innovative Synthesis and Tailored Design of HELHs

The emergence of HELHs represents a pivotal advancement in the domain of advanced materials, presenting promising prospects for diverse applications owing to their distinctive properties. However, the intricate nature of multi-component mixing poses substantial challenges in attaining universal stability and developing scalable synthesis methods for high-entropy compounds with varied compositions and structures.

### Controllable Synthesis for Ultrathin HELH Structures

In 2020, HELHs were first reported [45], with Mg^2+^, Al^3+^, Co^2+^, Ni^2+^, and Zn^2+^ ions uniformly distributed on two-dimensional sheets. These materials exhibited moderate thermal stability and hydroxide ion conductivity, thereby paving the way for the exploration of novel two-dimensional high-entropy materials. The innovative synthesis techniques such as advanced hydrothermal and framework-guided approaches have been pivotal in enhancing their structural, electrochemical, and catalytic properties.

**Fig. 3:**
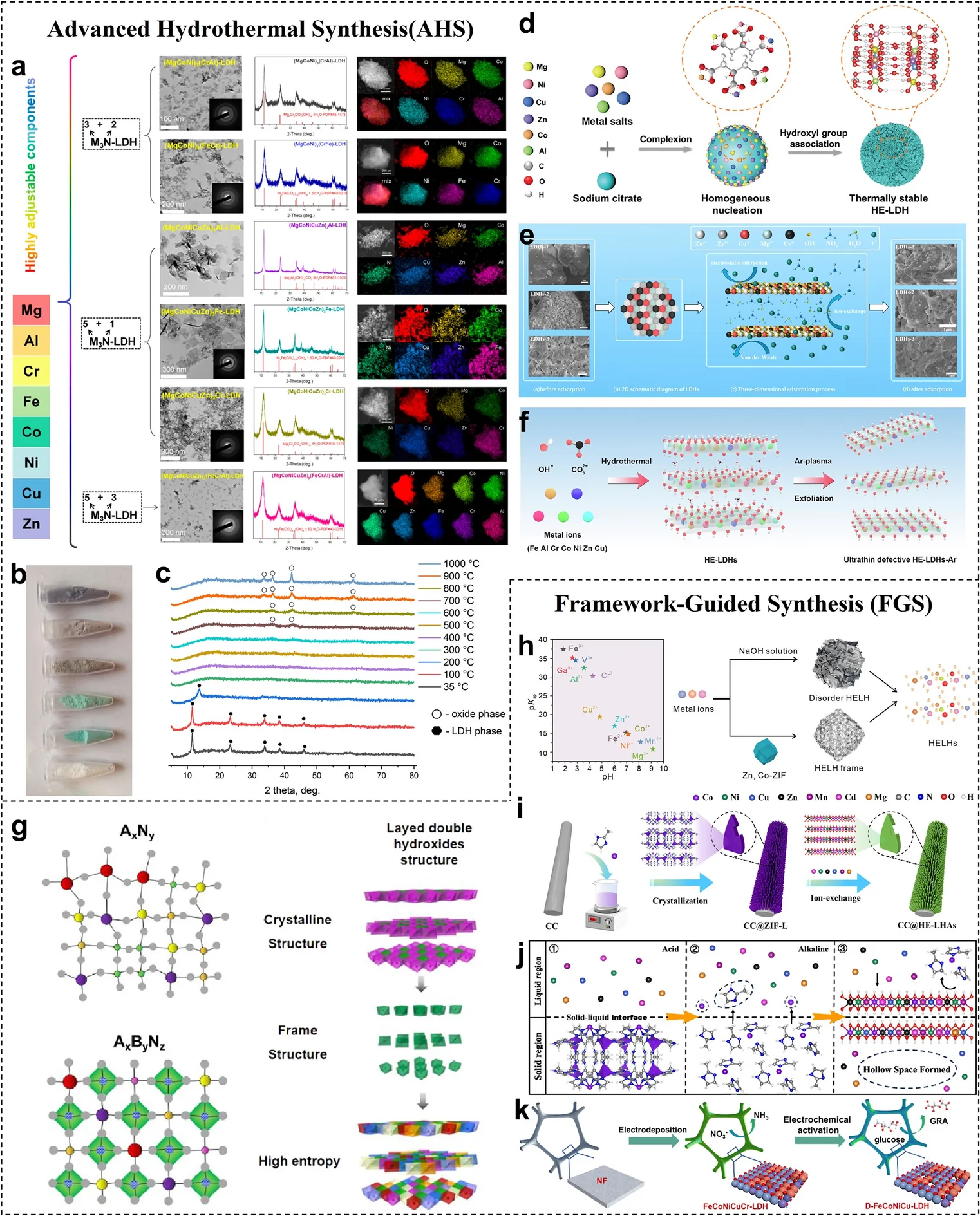
Advanced hydrothermal and framework-guided synthesis of HELHs.** a** Characterization of HELHs with various component combinations including TEM images, SAED images, XRD patterns, and elemental mapping for (MgCoNi)_3_(CrAl)-LDH, (MgCoNi)_3_(CrFe)-LDH, (MgCoNiCuZn)_3_Al-LDH, (MgCoNiCuZn)_3_Fe-LDH, (MgCoNiCuZn)_3_Cr-LDH, (MgCoNiCuZn)_3_(FeCrAl)-LDH. Reproduced with permission [46] Copyright 2023, American Chemical Society. **b** Pictures of LDHs powders, from bottom to top: Mg/Al, Mg/Ni/Al, MgNi/AlIn, MgNiCo/AlIn, MgNiCo/AlInSc, and MgNiCo/AlInScTm. **c** High-temperature X-ray diffraction of MgNiCo/AlInSc. Reproduced with permission [47] Copyright 2025, Royal Society of Chemistry. **d** Schematic diagram of the formation of HELHs. Reproduced with permission [48] Copyright 2024, Wiley. **e** Adsorption mechanism diagram of F^−^ by high-entropy ZnCoCrMgZr-LDHs. Reproduced with permission [49] Copyright 2024, Elsevier. **f** Illustration of the preparation of HELHs and ultrathin defective HELHs. Reproduced with permission [50] Copyright 2021, Elsevier. **g** Schematic diagram of distorted ion arrangement in high-entropy materials for metal compounds with the single metallic site (A_*x*_N_*y*_) and bimetallic sites (A_*x*_B_*y*_N_*z*_). And a schematic diagram of structural framework design in high-entropy layered double hydroxides structure. Green polyhedrons represent the coordination polyhedron of frame elements in the B site of A_*x*_B_*y*_N_*z*_, and the polyhedrons with other colors represent the coordination polyhedron of different metal elements as high-entropy components at the A site for A_*x*_B_*y*_N_*z*_. Reproduced with permission [51] Copyright 2022, American Chemical Society. **h** Illustration of the monolayer HELH frame preparation showing the p*K*_sp_ and the pH value at which ions (0.1 mol L^−1^) begin to precipitate. And a schematic diagram of synthesis of HELHs through co-deposition and in situ conversion from ZIF. Reproduced with permission [52] Copyright 2023, Wiley. **i** Schematic illustration of the synthesis process of self-supported hollow HE-LHAs on CC (named CC@LHAs(*n*)). **j** Kirkendall effect assisted ion exchange mechanism in the synthesis of hollow HE-LHA(7). Reproduced with permission [53] Copyright 2024, American Chemical Society. **k** Schematic for the synthesis of D-FeCoNiCu-LDH/NF. Reproduced with permission [54] Copyright 2024, Royal Society of Chemistry

Traditional catalysts frequently encounter limitations in compositional regulation, thereby impeding performance optimization. The combination of co-precipitation with pH control and hydrothermal treatment offers high universality in the synthesis of HELHs. Following this preparation strategy, Wu et al. [46] successfully synthesized a series of HELHs with varying component numbers and compositions (Fig. 3a). Some common metal elements used in LDH materials synthesis, including Mg^2+^, Al^3+^, Cr^3+^, Fe^3+^, Co^2+^, Ni^2+^, Cu^2+^, and Zn^2+^, were selected. Subsequently, a series of HELHs with varying component numbers and combinations were designed and synthesized, including quinary M_3_N-LDHs with three M^2+^ and two N^3+^ ions, senary M_3_N-LDHs with five M^2+^ and one N^3+^ ion, and even octadic M_3_N-LDHs with five M^2+^ and three N^3+^ ions. The specific HELHs compositions synthesized include (MgCoNi)_3_(CrAl)-LDH, (MgCoNi)_3_(CrFe)-LDH, (MgCoNiCuZn)_3_Al-LDH, (MgCoNiCuZn)_3_Fe-LDH, (MgCoNiCuZn)_3_Cr-LDH, and (MgCoNiCuZn)_3_(FeCrAl)-LDH. Basic characterization techniques, including transmission electron microscopy (TEM), X-ray diffraction (XRD), and elemental mapping at different magnifications, confirm the successful synthesis of these HELHs. Notably, as the composition of HELHs changes, variations in morphology and crystallinity are observed. However, the XRD peak shape and element distribution confirm that all synthesized HELHs maintain structural integrity. Additionally, TEM images and XRD patterns indicate that Fe^3+^ and Al^3+^ improve crystallinity and promote the formation of flatter nanosheets, whereas Cr^3+^ reduces crystallinity and leads to the formation of a network-like structure in HELHs. Moreover, the hydrothermal temperature required for single-phase formation varies depending on the elemental composition. The presence of Al^3+^ increases the hydrothermal temperature, resulting in a synthesis temperature of 120 °C for (MgCoNi)_3_(CrAl)-LDH and (MgCoNiCuZn)_3_Al-LDH. In contrast, Fe^3+^ lowers the hydrothermal temperature.

In addition to hydrothermal techniques, mechanochemical methods have also been employed to synthesize HELHs with diverse compositions. A series of HELHs, including Mg/Al, MgNi/Al, and MgNiCo/AlInSc, were prepared through a mechanochemically complemented synthesis process followed by hydrothermal treatment [47]. All the samples (Fig. 3b) were synthesized using a method involving manual mechanochemical synthesis, followed by soft hydrothermal treatment and additional crystallization in a saturated alkali solution. A study of the thermal transformations of MgNiCo/AlInSc revealed that above 200 °C, the samples decompose through metastable dehydrated forms, ultimately forming nonstoichiometric high-entropy oxides (Fig. 3c).

To enhance the thermal stability of HELHs, a novel complexation-assisted nucleation strategy has been developed to precisely regulate the nucleation process of metal ions [48]. Citrate carboxyl groups coordinate with metal ions via oxygen atoms, forming complex precursors (Fig. 3d). This coordination effectively suppresses the premature precipitation of specific metal ions caused by differences in solubility products. During the initial stages of the hydrothermal reaction, both these complexes and free metal ions gradually nucleate in solution, with citrate complexes acting as nucleation centers. This mechanism promotes homogeneous nucleation, mitigates phase segregation, and significantly enhances the high-temperature stability of HELHs. As a result, the synthesized materials exhibit exceptional thermal stability up to 300 °C, significantly outperforming conventional LDHs.

Achieving well-defined hydroxyl group formation is crucial for the successful synthesis of HELHs and their subsequent adsorption performance. To enhance the formation of hydroxyl groups essential for LDHs synthesis, a series of high-entropy ZnCoCrMgZr-LDHs was synthesized via a simple hydrothermal method, using triethanolamine (TEA) as the alkali source [49]. The incorporation of TEA also improved the adsorption capacity for fluoride ions (F⁻). The synthesized HELHs exhibited excellent fluoride adsorption performance. After adsorption, the block-like morphology of the LDHs remained unchanged. However, the surface became smoother, likely due to F⁻ occupying active sites on the adsorbent surface (Fig. 3e). The materials demonstrated strong resistance to anionic interference and good reusability, highlighting their potential for water treatment applications.

Among the various strategies explored, plasma etching has proven to be a highly effective method for synthesizing ultrathin HELHs with abundant structural defects. As demonstrated by Gu et al. [50] (Fig. 3f), this approach successfully generated defect-rich HELH nanosheets. The introduction of these structural defects played a crucial role in enhancing catalytic activity, thereby expanding the possibilities for designing HELHs with optimized properties for electrocatalytic applications.

Moreover, framework-guided synthesis has emerged as a highly effective strategy for designing high-entropy materials with tailored compositions and enhanced functionalities. By carefully stabilizing multiple metal components within a well-defined structural framework, this approach facilitates the formation of complex multi-metal compounds that exhibit superior stability and catalytic properties.

One major advantage of the framework-guided approach is its ability to stabilize diverse metal elements within a single-phase structure, where each metal site reinforces the others. Wu et al. [51] proposed a strategy for synthesizing high-entropy materials (Fig. 3g), addressing the challenge of achieving random metal element arrangement at low temperatures in single-metal-site compounds (A_x_N_y_). To overcome this, they designed a synthesis method based on bimetallic sites (A_x_B_y_N_z_), where significant differences in properties—such as ionic radius, coordination, and valence states—force the metal elements into a specific arrangement, thus preventing aggregation and reducing local stress. The strategy has been successfully applied in the synthesis of high-entropy perovskite hydroxides, layered double hydroxides, and spinel sulfides, all of which have shown considerable promise as electrocatalysts.

Furthermore, the synthesis of monolayer HELH frameworks under mild conditions has emerged as a promising solution. The solubility product (K_sp_) is commonly used to describe the precipitation sequence of metal ions, with a significant difference in pK_sp_ values indicating that the pH required for the formation of hydroxides varies greatly between ions. To achieve the monolayer HELH framework, Ding et al. [52] employed a Zn, Co-ZIF solution instead of traditional alkali liquor (Fig. 3h). In this approach, the organic ligands in ZIF are weakly bound to the metal ions, allowing the ZIF to decompose easily in an aqueous solution. As a result, ZIF continuously consumes H⁺ ions produced by the hydrolysis of the added ions, while the liberated metal ions from the original ZIF rapidly combine with these hydrolyzed ions, facilitating the formation of HELHs. The mild reaction conditions enable precise control over the structural and elemental composition, leading to HELHs with high surface areas that exhibit significant improvements in stability, conductivity, and active site availability.

Additionally, a novel metal–organic framework (MOF)-templated strategy was employed to construct hollow high-entropy layered hydroxide arrays (HE-LHAs) with multiple metal components [53]. The HE-LHAs were successfully synthesized through a simple two-step method involving crystallization and ion exchange (Fig. 3i). Uniform cobalt-based, leaf-like ZIF-L arrays with smooth surfaces were first directly grown on a flexible carbon cloth (CC) substrate. Subsequently, by immersing the CC@ZIF-L in ethanolic solutions containing various metal salts (Ni^2+^, Cu^2+^, Zn^2+^, Mn^2+^, Cd^2+^, and Mg^2+^), three-dimensional self-supported hollow arrays containing quinary, senary, or septenary HE-LHAs were formed on the CC surface. The formation of hollow arrays occurs through a dissolution-regrowth process driven by the classical Kirkendall effect, where the regrowth rate is slower than the dissolution rate (Fig. 3j). The acid-labile ZIF-L precursor decomposes in the weakly acidic ethanolic solution formed by the hydrolysis of metal salts. With the consumption of H^+^ ions, the framework of ZIF-L is decomposed, and thus, free Co^2+^ ions and 2-methylimidazole molecules are released on the sold–liquid interface, which can lead to the increase of the local pH value. Finally, the metal ions in solution as well as the Co^2+^ ions on the ZIF-L surface can co-precipitate with the OH^–^ ions. The hollow array structure and high-entropy composition significantly enhanced the catalytic activity. More importantly, the strategy overcomes the challenge of incorporating metal ions with large atomic radius differences, enabling even Cd^2+^ ions from the fourth period to be successfully incorporated into the lattice of the hollow LHAs.

Metallic mesh materials have also been employed as substrate frameworks for the synthesis of HELHs. For instance, Wu et al. [54] synthesized defect-rich, high-entropy FeCoNiCu LDH nanosheets grown on nickel foam (Fig. 3k). Initially, FeCoNiCuCr-LDH was synthesized on Ni foam via electrodeposition. The ultrathin FeCoNiCuCr-LDH nanosheets were vertically electrodeposited on the Ni foam, forming an open 3D structure. The electrochemical activation of FeCoNiCuCr-LDH/NF etched the Cr element, resulting in the formation of defect-rich FeCoNiCu-LDH/NF. The resulting D-FeCoNiCu-LDH/NF exhibited a robust, multi-site synergistic catalytic activity with tandem active sites, making it a promising catalyst.

The synthesis of high-entropy nanomaterials represents a rapidly advancing field, in which diverse methods are being investigated to precisely control their unique properties and optimize performance for targeted applications. As shown in Table 1, each synthesis approach exhibits distinct advantages and limitations. For example, hydrothermal and co-precipitation methods are widely employed due to their simplicity, cost-effectiveness, and scalability. Hydrothermal synthesis, in particularly, enables tunable morphology and high crystallinity. However, these methods are often constrained by limited morphological control, non-uniform elemental distribution, prolonged processing times, and the requirement for high temperatures and pressures. In contrast, plasma-assisted synthesis and electrodeposition offer superior precision in controlling material properties. Plasma-assisted synthesis is characterized by its rapid processing kinetics and ability to generate a higher density of active sites. Electrodeposition enables the direct growth of HELHs on conductive substrates, making it suitable for electrode fabrication. Ultimately, the selection of a synthesis method for HELHs depends on the target material properties and intended application. Researchers must carefully balance the benefits of improved performance metrics, such as reduced overpotential and lower Tafel slope, against practical limitations including cost, scalability, and control over morphology and composition. While these approaches have enabled precise control over structural and elemental compositions, resulting in enhanced performance, their industrial scalability remains a critical challenge. For instance, techniques such as plasma-assisted synthesis and MOF-templated methods demonstrate significant promise by providing exceptional control over morphology and composition. However, they encounter substantial barriers to large-scale production. Plasma-assisted synthesis typically requires specialized and energy-intensive equipment, rendering it less cost-effective for mass production. Its batch-based operation mode and stringent demands for precise parameter control further hinder scalability. Similarly, MOF-templated methods, although capable of producing highly porous and structurally well-defined materials, depend on complex, multi-step procedures and expensive organic ligands, making large-scale synthesis economically impractical. The recovery and recycling of these templating agents also remain challenging. Therefore, future research should prioritize the development of scalable and cost-efficient synthetic routes, such as continuous flow synthesis or enhanced hydrothermal approaches that employ abundant and low-cost precursors. Importantly, to accelerate the development and advancement of these technologies, we propose that future studies should provide detailed and transparent reporting of synthesis parameters and precursor costs. Such documentation would enable the research community to assess the practicality and cost-effectiveness of different synthetic approaches accurately, thereby facilitating collaborative progress, including the integration of artificial intelligence (AI) and machine learning (ML) for high-throughput screening and optimization of synthesis conditions. Addressing these challenges is essential for transitioning HELHs from laboratory-scale innovations into commercially viable materials.

**Table 1:** Summary of synthesis methods for HELHs

Synthesis method	Process conditions	Advantages	Disadvantages	Performance metrics	References
Hydrothermal	Precursor Concentration: Equimolar amounts (0.45 mmol) Reaction Volume: 40 mL DI water, 10 mmol urea Conditions: 180 °C, 12 h, in an autoclave	Tunable morphology, high crystallinity	High temp./pressure, time-consuming	η_10_ = 185 mV Tafel slope 49.7 mV dec^−1^	[55]
Coprecipitation	Precursors: A mixture of metal precursors dissolved in 25 mL of water Precipitating Agent: A solution of 0.6 g NaOH and 1.59 g Na_2_CO_3_. The pH was adjusted to 10	Simple, low-cost, scalable	Limited morphology control, less uniform element distribution	η_10_ = 230 mV 49.3 mV dec^−1^	[46]
Plasma-assisted	Material: 50 mg of hydrothermally synthesized HElHs Reactor: Plasma reactor using a quartz boat Conditions: Argon (Ar) plasma treatment for 20 min at 200 W	Etching treatment for enhanced active sites	Complex equipment, higher cost	η_10_ = 330 mV Tafel slope 63.7 mV dec^−1^ Double layer capacitances 0.33 mF cm^−2^	[50]
MOF-templated	Precursors: A combination of five different metal nitrates. Methanol and 2-MIM are used as the solvent and organic ligand, respectively Process: Metal ions first form MOF with 2-MIM, which then undergo a hydrolysis process during hydrothermal treatment to form the HELHs (120 °C for 4 h)	High surface area, porous 3D structures	Complex process, high MOF cost	η_10_ = 295 mV 61.79 mV dec^−1^ 5.1 mF cm^−2^	[56]
Electrodeposition	Precursors: A mixture of various metal sulfates and a molybdate. The electrolyte also contained 0.1 M Na_3_C_6_H_5_O_7_ and 0.15 g L^−1^ SDS as additives Process: The catalyst was electrodeposited using a square pulse potential mode at 25 °C in a three-electrode system. The process consisted of 3000 cycles, with an upper potential of 0 V and a pulse duration of 0.1 s, and a lower potential of − 2 V with a pulse duration of 0.1 s	Direct growth on substrate, ideal for electrodes	Requires conductive substrate, limited scalability	η_10_ = 248 mV 30 mV dec^−1^ 2.35 mF cm^−2^	[57]

Overall, the synthesis of HELHs has made significant advancements through innovative strategies such as hydrothermal, co-precipitation, mechanochemical, and framework-guided synthesis methods. These approaches have enabled refined control over structural and elemental compositions, as well as improved catalytic performance. However, challenges such as poor thermal stability, structural controllability, and limited active site availability remain. Therefore, continued development and improvement of efficient synthesis methods are essential. Although the use of MOF templates in synthesis effectively prevents phase separation and element aggregation, there is still a need to propose universal synthesis strategies that facilitate the large-scale production of HELHs. Additionally, these strategies should aim to reduce costs, simplify the process, and ensure scalability for practical applications.

### HELHs as Precursors for Advanced High-Entropy Materials

HELHs have emerged as promising precursors for the synthesis of HEOs and HESes, offering distinct advantages over conventional methods. Traditional synthesis approaches, such as high-temperature liquid phase synthesis, spray pyrolysis, and solution combustion, typically require elevated temperatures, which can induce phase separation and elemental aggregation, thereby compromising the uniformity and performance of the resulting materials. In contrast, utilizing HELHs as precursors offers the potential to synthesize HEOs and other high-entropy materials under milder conditions, effectively mitigating these issues.

**Fig. 4:**
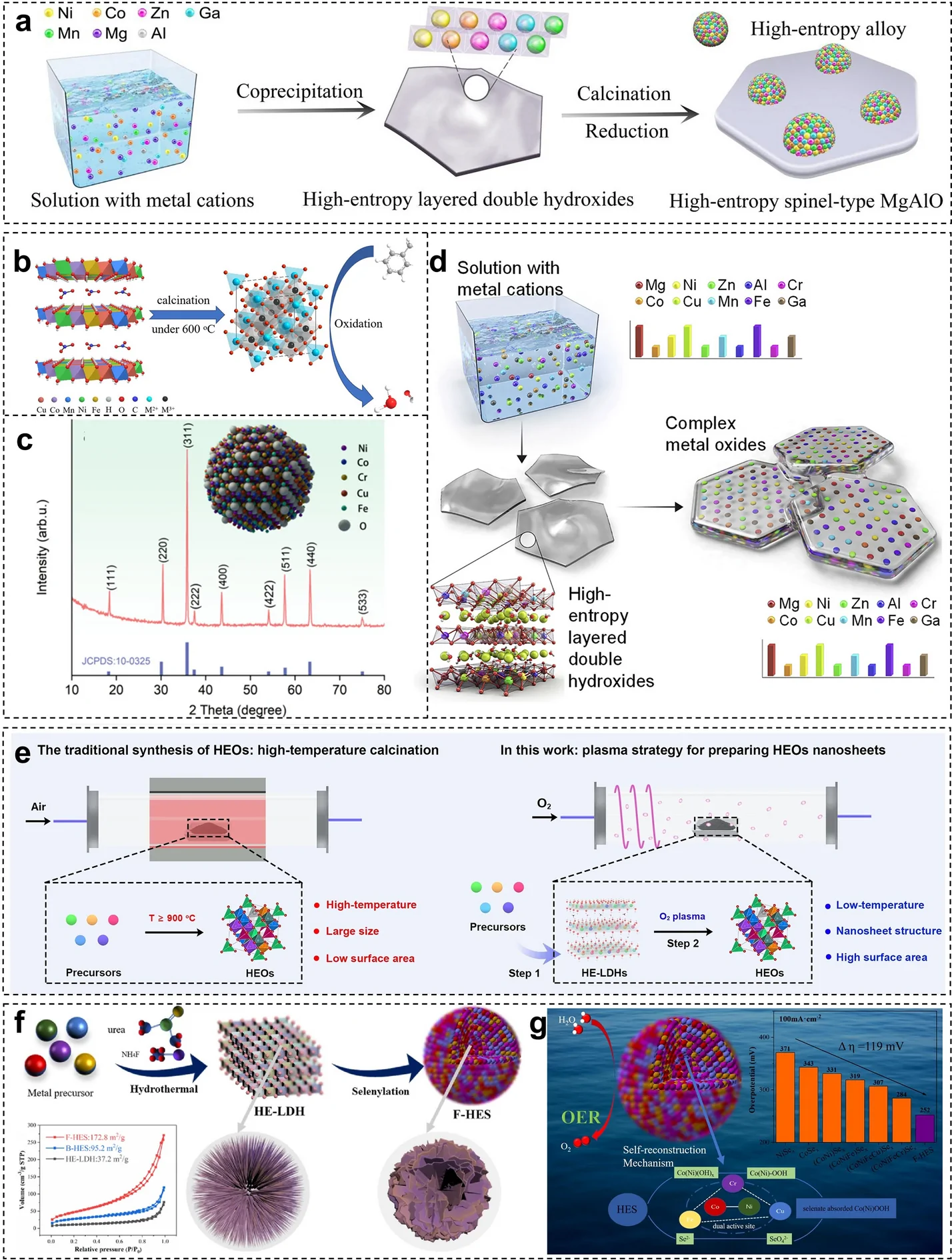
HELHs as precursors for the synthesis of high-entropy materials. **a** Schematic of porous high-entropy spinel-type HE-MgAlO (with up to five metals: Ni, Co, Zn, Ga, and Mn) catalysts derived from LDHs prepared using a one-step co-precipitation method. Reproduced with permission [58] Copyright 2024, Elsevier. **b** Schematic illustration of the preparation of high-entropy oxides (HEO) using multivariate composition LDHs as precursors. Reproduced with permission [60] Copyright 2023, MDPI. **c** XRD pattern of the high-entropy oxide (HEO), with an inset showing the schematic of HEO particle synthesis using HELHs as the precursor. Reproduced with permission [59] Copyright 2022, American Chemical Society. **d** Schematic illustration of HELHs and complex metal oxides. Reproduced with permission [61] Copyright 2022, Elsevier. **e** Schematic of the traditional synthesis method and low-temperature plasma strategy for high-entropy oxides (HEOs). Reproduced with permission [63] Copyright 2021, Wiley. **f** Schematic illustration of the preparation of flower-like high-entropy selenide (CoNiFeCuCr)Se (F-HES). Inset: N_2_ adsorption–desorption isotherms of as-prepared F-HES and bulk-like high-entropy selenide (B-HES). **g** F-HES catalyst shows excellent OER performance due to synergistic interactions among multiple metal species, with enhanced catalytic performance from high mixing entropy and lattice deformation. Reproduced with permission [25] Copyright 2023, Elsevier

Li et al. [58] demonstrated that high-entropy spinel-type MgAlO catalysts derived from HELHs exhibited enhanced catalytic activity and stability in biogas reforming reactions. The HE-MgAlO catalyst was synthesized by a one-step co-precipitation method to obtain the HELHs, which was then calcined at 700 °C for 4 h with a heating rate of 5 °C min^−1^ (Fig. 4a). The resulting catalyst, with its spinel structure and strong metal–support interactions, showed significantly improved thermal stability and exhibited excellent anti-sintering performance.

Similarly, Bushira et al. [59] reported that HELHs underwent significant changes in its crystal structure after calcination. XRD patterns confirmed the detachment of HELHs, leading to the disappearance of the electrostatic interface. This process resulted in the formation of defect-rich HEO (Fig. 4b). Furthermore, Xue et al. [60] found that thermal treatment at temperatures above 600 °C resulted in the complete transformation of HELHs into a single spinel-phase HEO (Fig. 4c). The optimized HEO catalyst demonstrated remarkable activity and stability in the catalytic oxidation of toluene. Kim et al. [61] also found that spinel-based complex oxides can be synthesized by heat treating the HELHs in air (Fig. 4d), with their metal cation ratios remaining stable even at elevated heat-treatment temperatures (up to 1200 °C). Besides, high-entropy layered metal oxides (HELMO) were designed by Wang et al. [62] to enhance the hydrogen storage performance of magnesium hydride (MgH_2_). By regulating the anionic intercalation (NO_3_/Cl) within high-entropy layered metal hydroxide precursors, the catalytic effect of HELMO on MgH_2_ was significantly improved. Specifically, HELMO-NO_3_/Cl was obtained by calcining HELMH-NO_3_/Cl at 500 °C under an argon atmosphere for 4 h at a heating rate of 2 °C min^−1^.

In contrast, Wang and colleagues [63] reported a low-temperature plasma strategy for synthesizing HEOs nanosheets with abundant oxygen vacancies (Fig. 4e). Plasma technology, promising for nanomaterial synthesis and modification, involves non-elastic collisions between high-energy electrons and oxygen molecules, transferring energy to the latter and generating chemically active oxygen species with significantly higher reactivity than molecular oxygen. HELHs, as precursors, can be oxidized by highly reactive oxygen species under mild conditions, transforming into single-phase spinel-type HEOs. The low-temperature plasma technique imparts nanosheet structures, abundant oxygen vacancies, and a high specific surface area to the synthesized HEOs, providing distinct advantages.

Traditional synthesis methods predominantly involve high-temperature processes, limiting control over shape and size, often yielding micron-scale materials with smaller surface areas. Due to the limited and inherently fewer active sites exposed by HESs, catalytic activity is often unsatisfactory. In this context, a two-step solvothermal method was utilized by Jiang et al. [25] to successfully synthesize flower-shaped high-entropy selenide (CoNiFeCuCr)Se (F-HES), which exhibited a superior specific surface area and demonstrated exceptional OER activity (Fig. 4f, g). Initially, various non-noble metal nitrates (iron, cobalt, nickel, copper, and chromium) were coordinated in proportion with urea, forming metal carbonate hydroxides. NH_4_F served as an etching agent, guiding growth along specific crystal directions to form sea urchin-like structures of HELHs. Subsequently, using HELHs as a template, ion exchange selenization transformed it into a single-phase F-HES. This unique preparation method offers a novel and effective approach for synthesizing high-entropy compounds.

To sum up, the use of HELHs as precursors for synthesizing HEOs and HESes offers notable advantages, including enhanced catalytic performance, stability, and scalability, achieved under milder synthesis conditions. This approach effectively addresses the limitations associated with traditional high-temperature synthesis methods. However, further research is needed to optimize synthesis methods, understand the complex interactions between different metal components during transformation, and improve the intrinsic activity and stability of the derived materials.

## Catalytic Superiority of HELHs: Mechanistic Insights and Performance Breakthroughs

### HELHs for OER: From Electronic Structure to Reaction Pathways

The OER represents a critical bottleneck in water electrolysis due to its sluggish kinetics, necessitating the development of efficient electrocatalysts. The concept of HEMs has garnered significant attention in electrocatalysis, as the incorporation of multiple metal cations can modulate electronic structures, enhance active site availability, and optimize adsorption energy with intermediates. This tunability is crucial for controlling the two primary OER mechanisms: the adsorbate evolution mechanism (AEM) and the lattice oxygen mechanism (LOM). The AEM is the conventional pathway where oxygen molecules are formed from water-derived intermediates on surface-active metal centers. This process is limited by a scaling relationship, which constrains catalytic activity [64, 65]. In contrast, the LOM bypasses this limitation by directly involving oxygen from the catalyst’s crystal lattice [66]. While the LOM can significantly lower overpotentials and improve kinetics, it often compromises long-term stability due to structural degradation [64]. A fundamental difference between these two mechanisms lies in their redox centers: AEM is a metal-redox process, while LOM is an oxygen-redox process [66]. As both mechanisms can occur simultaneously, OER electrocatalyst design faces a critical trade-off between high activity and long-term stability [64]. A variety of entropy-engineered materials, such as HEOs [67–69], HE-MOFs [70–72], and medium- to high-entropy alloys [73–75], have been demonstrated to exhibit excellent OER activity, highlighting the broad applicability of entropy modulation strategies in catalyst design. HELHs leverage the synergistic effects of entropy stabilization, lattice distortion, and multi-metal electronic coupling to achieve superior OER performance.

**Fig. 5:**
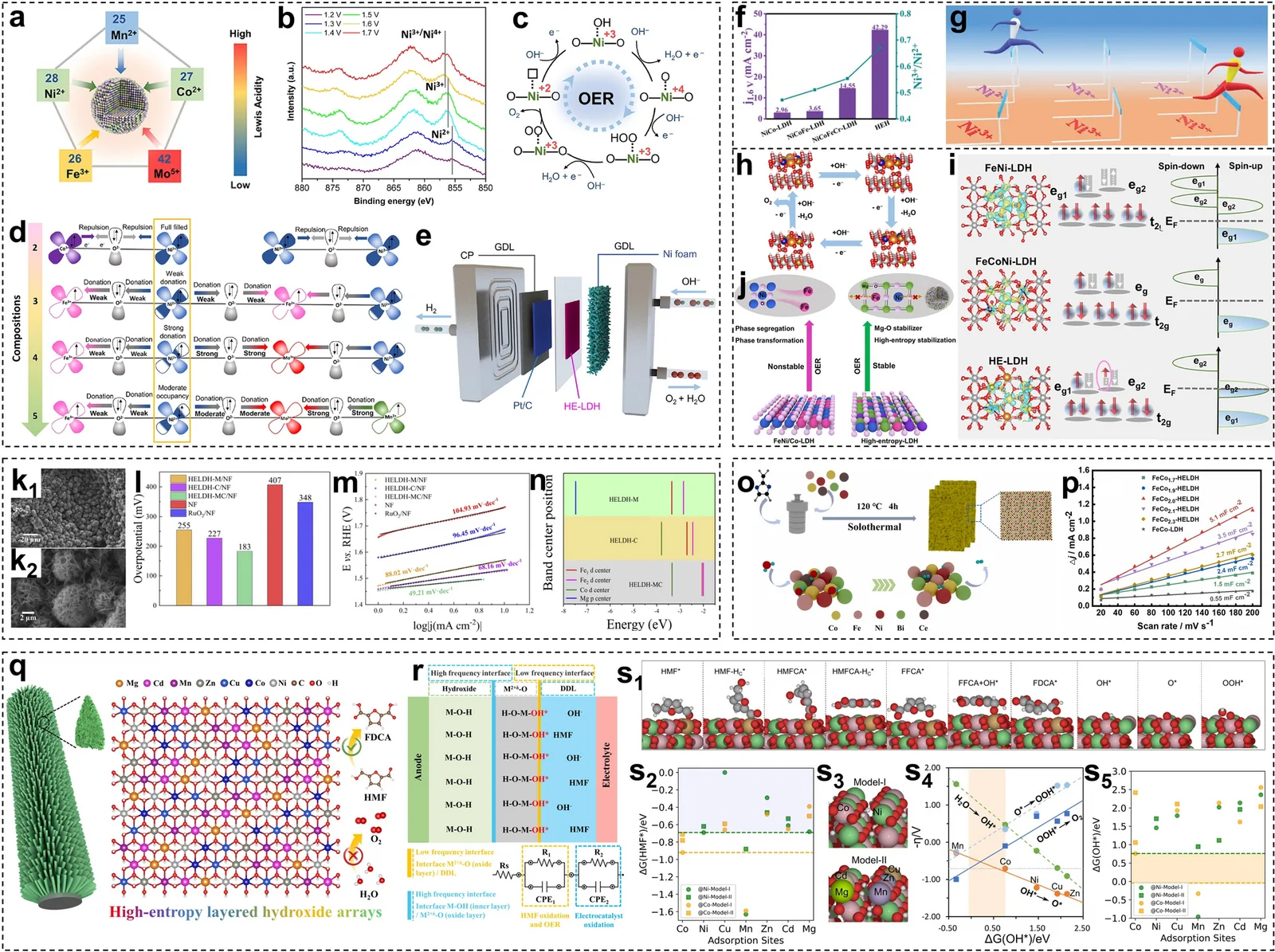
Structural, electronic, and electrochemical properties of HELHs for OER applications. **a** Elemental composition of the HELHs. **b** Ni 2*p* XPS spectra of high-entropy LDHs under different applied potentials during the OER process. **c** Schematic illustration of the valence state transition of active Ni in response to different adsorbed intermediates involved in the OER via the AEM pathway. **d** Schematic representation of the interplay between compositional 3d orbitals through metal–oxygen–metal bonds in LDHs and their derived oxyhydroxides. **e** Schematic diagram of an AEM water electrolysis device (CP: carbon paper; GDL: gas diffusion layer; AEM: anion exchange membrane). Reproduced with permission [78] Copyright 2024, Elsevier. **f** Correlation between current density at 1.6 V and the Ni^3+^/Ni^2+^ ratio for different catalysts. **g** Schematic illustration of the beneficial role of high-valence Ni^3+^ in enhancing OER performance. Reproduced with permission [79] Copyright 2022, Royal Society of Chemistry. **h** Schematic representation of the OER process for HELHs. **i** Charge density difference and electron population distribution in the 3d orbitals of Ni atoms in the as-synthesized LDHs. **j** Schematic illustration of structural evolution during the OER process for FeCoNi-LDH and high-entropy LDH. Reproduced with permission [80] Copyright 2023, American Chemical Society. **k1**, **k2** SEM images of HELHs-MC/NF at low and high magnifications, respectively. **l** Overpotentials at a current density of 10 mA cm^−2^. **m** Tafel plots of the optimal HELH-M/NF, HELH-C/NF, HELH-MC/NF, bare NF, and RuO_2_/NF. **n** d-band center positions of Fe and Co atoms, as well as the p-band center of Mg, in three high-entropy materials. Reproduced with permission. [82] Copyright 2023, Royal Society of Chemistry. **o** Schematic illustration of the synthesis process for FeCo_x_-HELHs nanosheets. **p** Electrochemical double-layer capacitance of FeCo_x_-HELHs. Reproduced with permission. [56] Copyright 2024, Elsevier. **q** Schematic diagram of self-supported hollow HE-LHAs on CC (denoted as CC@LHAs(n)), which enhance 5-hydroxymethylfurfural (HMF) electrooxidation by suppressing oxygen evolution. **r** Schematic representation of the relationships between surface hydroxide/hydroxyl oxide species, interfaces, and reactants (H_2_O and HMF). **s1** Representative adsorption structures of reaction intermediates on (Cu, Co, Ni)–OOH and (Co, Ni)–OOH surfaces. **s2** Free energy change (ΔG) of HMF* adsorption on active sites in models I and II. **s3** Molecular structures of models I and II. **s4** Activity volcano plot for OER based on model I. **s5** Free energy change (ΔG) of OH* adsorption at active sites in models I and II (@Co/Ni denotes either the Co/Ni site or a dopant at the Co/Ni site). Reproduced with permission [53] Copyright 2024, American Chemical Society

Nickel-based LDHs have emerged as promising OER catalysts [76, 77], yet they often suffer from limited activity and stability. The rational design of nickel-based HELHs by tuning configuration entropy has led to unprecedented OER activity. Liu et al. [78] demonstrated that introducing Lewis acidic cations into Ni-LDHs significantly enhances catalytic performance by modulating the electronic states of Ni active centers through cation–oxygen bridge bonds (Fig. 5a). In situ X-ray photoelectron spectroscopy (XPS) reveals that Ni in HELHs oxidizes from Ni^2+^ to Ni^3+^ at the OER onset potential and further to a mixed Ni^3+^/Ni^4+^ state at 1.6–1.7 V, indicating strong interactions with oxygen intermediates and dynamic valence changes during OER (Fig. 5b). Theoretical calculations show that the energy barriers for the potential determining step (PDS) in lattice oxygen mechanism (LOM) and oxide path mechanism (OPM) (0.83 and 0.55 eV, respectively) exceed that of the adsorbate evolution mechanism (AEM) pathway (0.33 eV), suggesting that AEM predominates in HELHs catalysts (Fig. 5c). Electronic interactions in binary and HE-FeCoNiMoMn-LDH catalysts (Fig. 5d) follow distinct charge transfer pathways. In the binary CoNi system, Coulomb repulsion between fully occupied t_2_g orbitals of Co^2+^/Ni^2+^ and O 2*p* orbitals limits electron transfer, keeping Ni in a lower valence state. In contrast, in HE-FeCoNiMoMn-LDH, Mn^2+^ donates electrons to Mo^5+^ and Fe^3+^ via π-donation with O^2−^, alleviating Ni electron depletion. An AEM electrolyzer with Pt/C for HER and HELHs for OER (Fig. 5e) demonstrated a low overpotential (203 mV at 10 mA cm^−2^) and exceptional durability, maintaining 1 A cm^−2^ at 1.73 V for over 100 h, underscoring the impact of electronic structure engineering on OER performance.

Similarly, Zhang et al. [79] proposed enhancing the catalytic activity of HELHs by inducing amorphization and maximizing high-valence Ni^3+^ content. The increasing turnover frequency (TOF) and OER current density align with the rising Ni^2+^Ni^3+^/Ni^2+^ ratio, highlighting the beneficial role of high-valence Ni species (Fig. 5f). The near-unity e_g_ occupancy of Ni^3+^ (t_2g_^6^ e_g_^1^) is known to lower the reaction barrier, thereby improving OER performance (Fig. 5g). Additionally, Liu et al. [80] introduced a high-entropy coordination strategy incorporating inert Mg to optimize OER performance. Figure 5h illustrates the adsorption configurations of OER intermediates. Ni 3d orbitals in HELHs exhibit an electron distribution between 3d^7^ and 3d^8^ due to the synergistic effects of Fe, Co, and Mg. This results in partial spin-up occupancy at the e_g2_ level, with one spin-up and two spin-down states remaining at the e_g_ level (Fig. 5i). Acting as electronic regulators, Fe, Co, and Mg cations collaboratively modulate the electronic structure of Ni, optimizing its electron state and enhancing OER activity. Besides, Mg-O interfacial bonds suppress phase segregation and structural degradation, ensuring long-term stability (Fig. 5j). Consequently, HELHs nanosheets exhibit both high activity (302 mV at 100 mA cm^−2^) and exceptional durability.

Interestingly, Li et al. [81] reported that CaFe–LDH, originally synthesized for the treatment of multi‐metal-contaminated wastewater, efficiently removed Co^2+^, Ni^2+^, Cu^2+^, and Zn^2+^. These metal ions were subsequently mineralized into CaCoNiCuZnFe‐HELH via isomorphic substitution of Ca^2+^ within the CaFe laminate. Combined XPS and X-ray absorption fine structure (XAFS) analyses revealed that the incorporation of cations with different ionic radii induced lattice distortions, which facilitated electron transfer from Co and Ni to Fe through Co/Ni–O–Fe linkages and thereby enhanced Co/Ni–O covalency. The resulting CaCoNiCuZnFe‐HELH exhibited outstanding OER activity, requiring an overpotential of 310.7 mV at 10 mA cm^−2^.

In addition to optimizing electronic properties, structural engineering of HELHs has proven to be an effective strategy for enhancing OER efficiency. Li et al. [82] demonstrated the synthesis of seven-element HELH microspheres using a hydrothermal method. The hierarchical structure, observed via scanning electron microscopy (SEM) (Fig. 5k), consists of interconnected nanosheets supported on a nickel foam substrate. This structure exhibited an ultra-low overpotential of 183 mV at 10 mA cm^−2^ (Fig. 5l) and a small Tafel slope of 49.21 mV dec^−1^ (Fig. 5m). In comparison with other catalysts, the shift in the d-band center of HELH-MC leads to the elevation of the antibonding orbital, facilitating the adsorption of intermediates on the catalyst surface (Fig. 5n). Likewise, high-entropy LDH FeNiCoMnCr, grown on nickel foam via hydrothermal synthesis [83], and FeCoNiMoZn-OH high-entropy hydroxide, synthesized by pulse electrochemical deposition [57], had been, respectively, reported to exhibit excellent OER activity in alkaline conditions.

As mentioned earlier, the synthesis of framework-directed HELH electrocatalysts has attracted considerable attention in recent years, with the MOF-mediated synthesis strategy emerging as a particularly promising approach that enables precise control over composition and morphology. Indeed, HELHs prepared via this facile MOF-mediated method (Fig. 5o) were reported by Chu et al. [56] to exhibit remarkable OER activity, with their superior performance attributed to optimized binding energies for intermediates and enhanced electron mobility. Based on CV curves at different scan rates (Fig. 5p), the double-layer capacitance (C_dl_) of FeCo2.0-HELH was determined to be 5.1 mF cm^−2^, significantly exceeding that of other materials. Additionally, Ding et al. [52] demonstrated an in situ conversion strategy to prepare a monolayer HELH frame structure. This unique morphology, which prevents layer aggregation and the formation of a local acidic environment, allowed the catalyst to reach 100 mA cm^−2^ at an overpotential of 259 mV in 1 M KOH and sustain 20 mA cm^−2^ for an impressive 1000 h with no obvious deterioration in catalytic performance. These findings validate the potential of framework-directed HELHs as next-generation OER catalysts. Similarly, ZIF nanocrystals have been employed as precursors to synthesize HELHs via a metal–organic framework-derived strategy [84]. The resulting CoZnFeNiAl-HELH catalyst exhibits remarkable OER performance, requiring an overpotential of only 276 mV at a current density of 100 mA cm^−2^.

Interestingly, HELHs have been explored not only for enhancing OER but also for suppressing it in favor of alternative oxidation reactions. Hollow HELH arrays (HE-LHAs) synthesized via a MOF-templated strategy have been shown to preferentially catalyze the oxidation of 5-hydroxymethylfurfural (HMF) [53], while minimizing OER competition (Fig. 5q). In situ EIS tests clarify the interfacial evolution of various electrocatalysts between the electrode and electrolyte (Fig. 5r). No HMF passivation is observed on CC@LHA(7), which also shows a high Faradaic efficiency of 99.99% for 2,5-furandicarboxylic acid (FDCA) by suppressing OER. Theoretical calculations reveal the typical adsorption structures of the intermediates (Fig. 5s1). The ΔG(HMF*) values for different doped sites in models I and II reveal that most sites fall into regions favorable for enhanced activity (Fig. 5s2, s3). The OER activity volcano plot for Co sites (doped with Mn, Cu, and Zn) indicates that when ΔG(OH*) is in the light orange region, OER activity can be further enhanced (Fig. 5s4). However, the ΔG(OH*) values of all active sites in models I and II deviate from the optimal region, suggesting that metal doping suppresses OER activity, thus enhancing the HMFOR activity and selectivity (Fig. 5s5).

### Robust Water Oxidation Catalysis via Dynamic Surface Reconstruction

Water splitting is a promising strategy for sustainable hydrogen production, yet the sluggish kinetics of the OER present a significant challenge. HELHs have emerged as a class of electrocatalysts with unique structural and electronic properties, offering enhanced OER activity and stability.

**Fig. 6:**
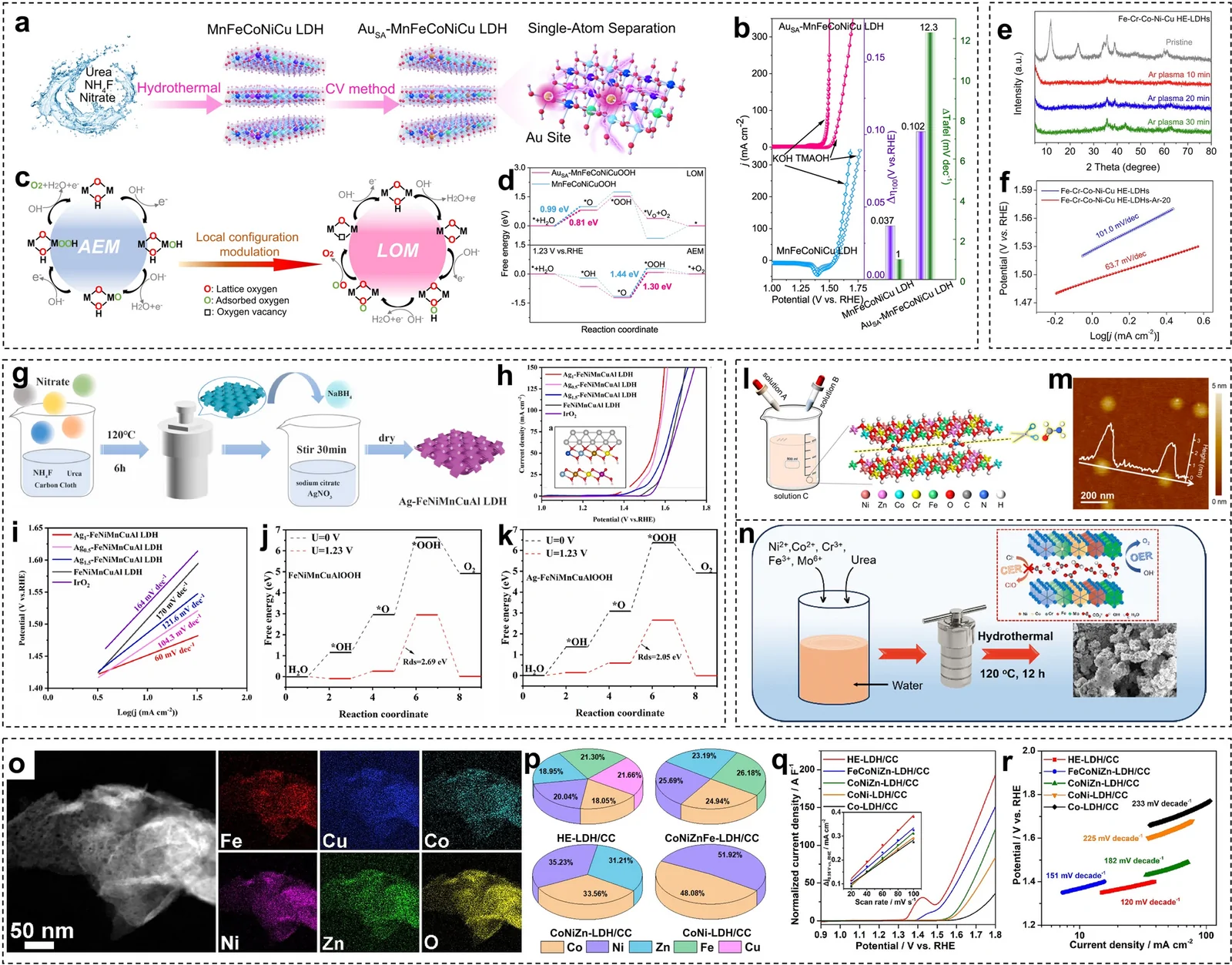
Multifunctional HELHs for robust water oxidation. **a** Synthesis schematic of Au_SA_-MnFeCoNiCu LDH. **b** Polarization curves of Au_SA_-MnFeCoNiCu LDH and MnFeCoNiCu LDH in 1.0 M KOH and 1.0 M TMAOH (left); shift of overpotential at 100 mA cm^−2^ (Δη_100_) and Tafel slopes from KOH to TMAOH (right). **c** AEM and LOM on MnFeCoNiCuOOH. **d** Computed free energy diagrams (ΔG) for OER steps on Au_SA_-MnFeCoNiCuOOH and MnFeCoNiCuOOH. Reproduced with permission [85] Copyright 2023, Springer Nature**. e** XRD patterns of Fe–Cr-Co–Ni-Cu HELHs treated with Ar plasma. **f** Tafel plots of Fe–Cr-Co–Ni-Cu HELHs and Fe–Cr-Co–Ni-Cu HELHs-Ar-20. Reproduced with permission [50] Copyright 2021, Elsevier. **g** Synthesis schematic of the Ag-FeNiMnCuAl LDH electrocatalysts. **h** Linear sweep voltammetry (LSV) curves (inset: structural model of Ag-FeNiMnCuAlOOH) and **i** Tafel plots of Ag_1_-FeNiMnCuAl LDH, Ag_0.5_-FeNiMnCuAl LDH, Ag_1.5_-FeNiMnCuAl LDH, FeNiMnCuAl LDH, and IrO_2_. Gibbs free energy of adsorption intermediates of **j** FeNiMnCuAlOOH and **k** Ag-FeNiMnCuAlOOH. Reproduced with permission [87] Copyright 2024, Elsevier. **l** Synthetic scheme of U-NiFeZnCoCr-LDH. **m** Tafel slopes of NiFe-LDH, NiFeZnCoCr-LDH, and U-NiFeZnCoCr-LDH. Reproduced with permission [90] Copyright 2024, MDPI. **n** Schematic illustration of the synthesis of high-entropy NiCrCoFeMo layered hydroxide (HELH) and its ability to promote sustainable seawater oxidation. Reproduced with permission [91] Copyright 2024, Royal Society of Chemistry. **o** High-angle annular dark-field scanning transmission electron microscopy (HAADF-STEM) image with elemental distribution mapping of HELH/CC. **p** ICP-OES results of HELH/CC and its medium-/low-entropy counterparts. **q** C_dl_-normalized OER activity with the calculation of C_dl_ values shown in the inset. **r** Tafel plots for evaluating the urea oxidation reaction (UOR) performance. Reproduced with permission [92] Copyright 2023, Elsevier

The oxygen evolution reaction is often limited by the conventional AEM. However, triggering the LOM can surpass this theoretical limit and accelerate reaction kinetics. Wang et al. [85] demonstrated that Au single atoms and oxygen vacancies incorporated into a MnFeCoNiCu layered double hydroxide (Au_SA_-MnFeCoNiCu LDH) significantly improved OER performance (Fig. 6a). The catalyst shows significantly reduced OER activity in 1.0 M TMAOH electrolyte relative to 1.0 M KOH electrolyte (Δη_100_ = 0.102 V, ΔTafel = 12.3 mV dec^−1^), implying the binding between TMA^+^ and O_2_^2−^ and validating that Au_SA_-MnFeCoNiCu LDH undergoes LOM during OER (Fig. 6b). Normally, AEM consists of four basic steps with three different intermediates (*OH, *O, and *OOH), while LOM involves five basic steps including four different intermediates (*O, *OOH, *OO, and V_O_) (Fig. 6c). DFT calculations revealed Au_SA_-MnFeCoNiCuOOH prefers to follow LOM relative to MnFeCoNiCuOOH, thereby triggering the LOM and boosting catalytic activity (Fig. 6d). Similarly, Yao et al. [86] designed FeCoNiCuZn LDHs with abundant cation vacancies, which exhibit low overpotentials of 227, 275, and 293 mV to reach 10, 100, and 200 mA cm^−2^, respectively. DFT calculations further confirm that these cation vacancies enhance the intrinsic activity of HELHs by optimizing the adsorption energy of OER intermediates.

In addition to element doping and vacancies, defect engineering provides an effective strategy for enhancing OER activity. A hydrothermally synthesized Fe–Cr-Co–Ni–Cu HELH was further treated with plasma etching, yielding ultrathin nanosheets with abundant defects [50]. This exfoliation process not only preserved the single-phase crystalline structure but also facilitated uniform atomic distribution. XRD patterns suggest that the HELHs are successfully exfoliated and plasma is capable of destroying the electrostatic interactions in HELHs (Fig. 6e). Tafel slope of Fe–Cr–Co–Ni–Cu HELHs-Ar-20 was calculated to be 63.7 mV dec^−1^, lower than that of Fe–Cr–Co–Ni–Cu HELHs (Fig. 6f).

Moreover, the construction of the novel high-entropy heterostructure catalyst has been explored to enhance conductivity and electronic interactions [87]. A FeNiMnCuAl LDH catalyst decorated with Ag nanoparticles was fabricated using hydrothermal and chemical reduction methods (Fig. 6g). The catalyst achieved an exceptionally low overpotential of 208 mV at 10 mA cm^−2^ (Fig. 6h) and a Tafel slope of 60 mV dec^−1^ (Fig. 6i). The Gibbs free energy profiles (Fig. 6j, k) reveal that the Ag nanoparticles contributed to improved electrical conductivity and optimized energy barriers, thereby enhancing the intrinsic OER activity.

Although HELHs exhibit excellent catalytic activity, their inadequate long-term stability remains a critical challenge. NiFe-based LDHs have demonstrated versatile electrocatalytic performance in both OER [88] and HER [89], serving as a robust platform for activity enhancement. Jing et al. [90] developed a rapid and facile bottom-up approach to synthesize ultrathin Ni–Co–Fe–Cr–Zn HELHs (Fig. 6l). Compared to conventional NiFe-LDH, the optimized HELH exhibited significantly a lower Tafel slope, indicating enhanced OER reaction kinetics (Fig. 6m). Furthermore, its performance decay was approximately eight times lower than that of NiFe-LDH, highlighting its superior durability.

The direct electrolysis of seawater presents additional challenges, primarily due to the competing chlorine evolution reaction. To address this, a novel NiCrCoFeMo HELH was synthesized using a single-step hydrothermal method, exhibiting superior catalytic performance compared to its quaternary and ternary counterparts (Fig. 6n) [91]. This enhancement can be attributed to the presence of a greater number of electroactive sites and an improved chloride-restricting capability of interlayer anions. Moreover, the formation of β-NiOOH during prolonged electrooxidation further contributed to the sustained catalytic activity. Notably, integrating this HELH with Pt/C in a two-electrode system achieved a high current density of 500 mA cm^−2^ at 1.92 V in a 6 M KOH seawater electrolyte, underscoring its potential for practical seawater electrolysis applications.

Beyond conventional OER electrocatalysis, HELHs have also been investigated for urea-assisted water electrolysis. Hao et al. [92] synthesized a lattice-disordered high-entropy FeCuCoNiZn hydroxide nanoarray catalyst grown on carbon cloth (HELH/CC), which exhibited outstanding bifunctional OER and urea oxidation reaction (UOR) activity. High-angle annular dark-field STEM (HAADF-STEM) images revealed the presence of abundant nanopores, while elemental mapping confirmed the homogeneous distribution of all metallic elements and oxygen across the nanosheets (Fig. 6o). Inductively coupled plasma optical emission spectroscopy (ICP-OES) analysis further demonstrated that HELH/CC contains nearly equal proportions of all metallic elements, underscoring its high-entropy characteristics (Fig. 6p). LSV measurements indicated that HELH/CC exhibited the highest OER activity among the tested samples (Fig. 6q). Moreover, it possessed a larger electrochemical double-layer capacitance of 1.66 mF cm^−2^ compared to medium-/low-entropy catalysts (1.14–1.40 mF cm^−2^), suggesting an increased electrochemically active surface area (ECSA) due to its lattice-disordered nanoporous structure. Additionally, HELH/CC exhibited a smaller Tafel slope of 120 mV dec^−1^ compared to its counterparts (Fig. 6r), indicating improved charge transfer kinetics. Furthermore, the catalyst demonstrated excellent stability in both OER and UOR, with significant performance enhancements of 35.3% and 88.7%, respectively, over long-term operation. Likewise, a catalyst composed of HELHs supported on multi-walled carbon nanotubes (FeAlNiCoMn-HELH/CNT) was proposed [93]. It was synthesized through a simple hydrothermal method, combining metal ion nitrate precursors with piranha‑treated multi‑wall carbon nanotubes (O‑MWCNT). The catalyst exhibited an overpotential of 243 mV at 10 mA cm^−2^ and a Tafel slope as low as 46.54 mV dec^−1^, demonstrating excellent catalytic performance for the OER. Additionally, HELH nanoneedles have been reported [55], comprising a cost‑effective mix of non‑precious transition metals (Fe, Co, Cr, Mn, Zn, denoted as FCCMZ). These FCCMZ HELH nanoneedles deliver a low overpotential of 185 mV at 10 mA cm^−2^ and a minimal Tafel slope of 49.7 mV dec^−1^, outperforming ternary and quaternary LDHs. For the UOR, the FCCMZ HELH achieves an exceptionally low potential of 250 mV.

**Fig. 7:**
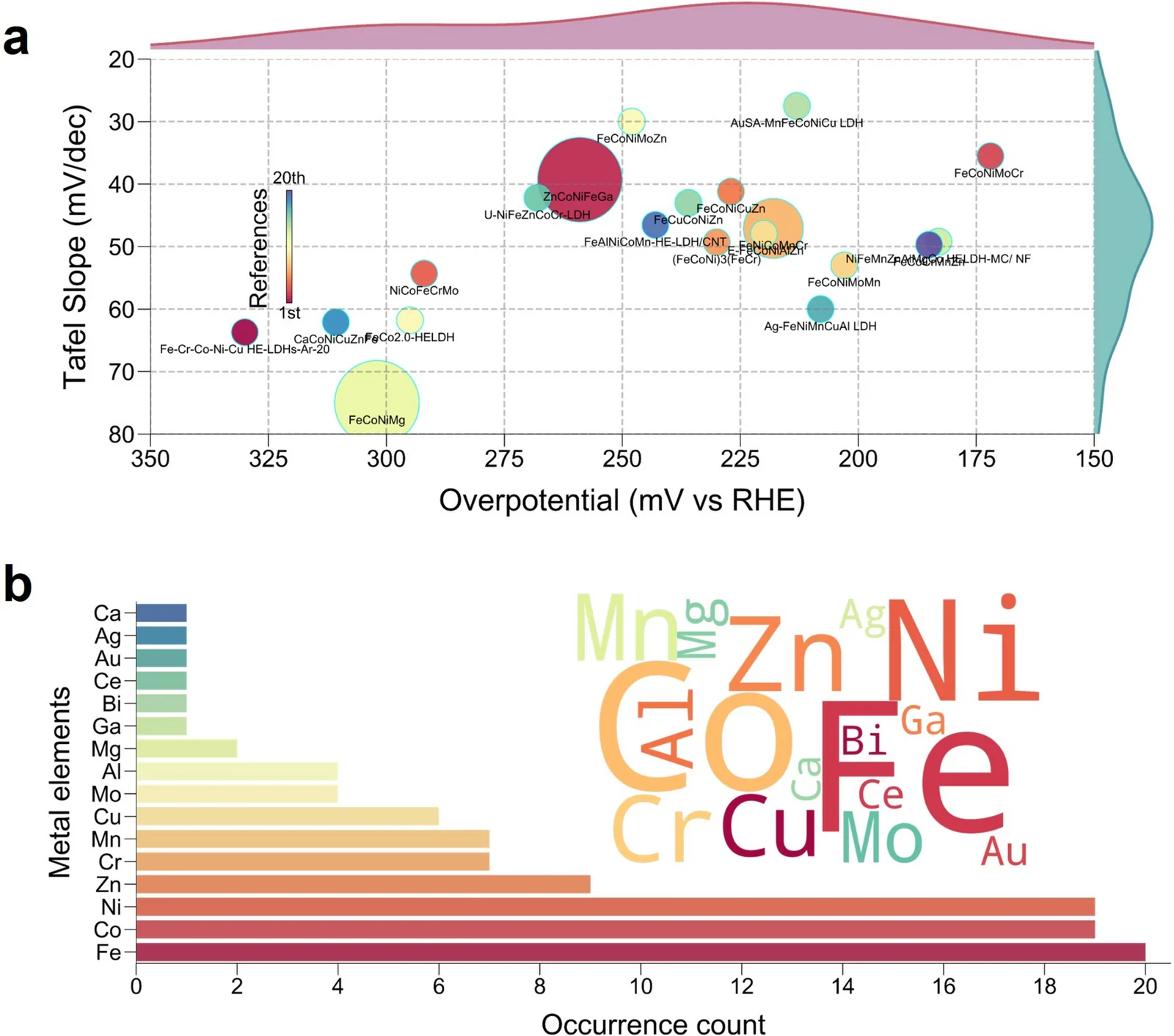
**a** Bubble chart of the OER activity of different HELHs. The data are derived from Table 2. The size of the circles indicates the current density, increasing in the order of 10, 50, and 100 mA cm^−2^. **b** The frequency of different metal elements in the HELHs catalysts listed in Table 2. A bar chart displays the count of each element, with a corresponding word cloud inset to visualize their frequency distribution

In general, as evidenced in Table 2, numerous studies have focused on synthesizing various HELHs to optimize their performance in the OER. A diverse range of synthetic methods has been employed, with hydrothermal, co-precipitation, and framework-guided synthesis being the most common. These OER performance tests were conducted under the standard 1 M KOH conditions. To visually assess the catalytic activity of these materials, the data from Table 2 are represented in a bubble chart as shown in Fig. 7a. Catalysts situated in the upper-right quadrant of the chart, characterized by low overpotentials and low Tafel slopes, demonstrate the most outstanding OER activity. To further investigate the source of HELHs’ high performance, a statistical analysis of the metal elements from Table 2 is presented in Fig. 7b, revealing the key elements and their frequency distribution. The bar chart and corresponding word cloud (inset of Fig. 7b) visually depict the frequency of different metal elements in reported systems. The results indicate that iron (Fe), cobalt (Co), and nickel (Ni) are the most frequently used and core elements for constructing high-performance OER catalysts. These are followed by zinc (Zn), chromium (Cr), manganese (Mn), and copper (Cu), with other elements appearing less frequently. The prevalence of these elements aligns with their critical roles as active centers or co-catalysts, where multi-metallic synergy is key to modulating electronic structures, optimizing the adsorption of reaction intermediates, and enhancing the overall stability of the catalysts. This systematic analysis of key elements provides valuable guidance for the rational design of future high-performance HELHs.

**Table 2:** Benchmarking the activities of various HELHs on OER performance

Catalysts	*η*	*j*	*b*	*C*_*dl*_	Metal elements	Synthesis method	Year	References
Fe–Cr–Co–Ni–Cu HELHs-Ar-20	330	10	63.7	0.33	Cr,Fe,Co,Ni,Cu	Plasma-Assisted hydrothermal	2021	[50]
ZnCoNiFeGa HELH	259	100	39.3	–	Fe,Co,Ni,Zn,Ga	in situ conversion	2023	[52]
FeCoNiMoCr HELH	172	10	35.53	6.93	Cr,Fe,Co,Ni,Mo	electrodeposition stacking	2024	[94]
NiCoFeCrMo	292	10	54.32	–	Cr,Fe,Co,Ni,Mo	hydrothermal	2022	[79]
FeCoNiCuZn	227	10	41.2	–	Fe,Co,Ni,Cu,Zn	hydrothermal	2023	[86]
(FeCoNi)_3_(FeCr)	230	10	49.3	–	Cr,Fe,Co,Ni	co-precipitation and hydrothermal	2023	[46]
FeNiCoMnCr	218	50	47.1	0.11	Cr,Mn,Fe,Co,Ni	hydrothermal	2023	[83]
FeCoNiMoMn	203	10	53	–	Mn,Fe,Co,Ni,Mo	template-assisted ions exchange	2024	[78]
E-FeCoNiAlZn	220	10	47.92	–	Al,Fe,Co,Ni,Zn	chemical reduction and etching	2025	[95]
FeCo2.0-HELH	295	10	61.79	5.1	Fe,Co,Ni,Ce,Bi	MOF-mediated synthesis	2024	[56]
FeCoNiMoZn	248	10	30	2.35	Fe,Co,Ni,Zn,Mo	pulsed electrochemical deposition	2024	[57]
FeCoNiMg	302	100	75	37	Mg,Fe,Co,Ni	hydrothermal	2023	[80]
NiFeMnZnAlMgCo HELH-MC/NF	183	10	49.21	80.64	Mg,Al,Mn,Fe,Co,Ni,Zn	hydrothermal	2023	[82]
Au_SA_-MnFeCoNiCu LDH	213	10	27.5	–	Mn,Fe,Co,Ni,Cu,Au	hydrothermal	2023	[85]
FeCuCoNiZn	236	10	43	1.66	Fe,Co,Ni,Cu,Zn	hydrothermal	2023	[92]
U-NiFeZnCoCr-LDH	268	10	42.2	1.08	Cr,Fe,Co,Ni,Zn	modified co-precipitation	2024	[90]
Ag-FeNiMnCuAl LDH	208	10	60	19.53	Al,Mn,Fe,Ni,Cu,Ag	hydrothermal and chemical reduction	2024	[87]
CaCoNiCuZnFe	310	10	62.1	0.21	Ca,Fe,Co,Ni,Cu,Zn	mineralization	2025	[81]
FeAlNiCoMn-HELH/CNT	243	10	45.54	6.21	Al,Mn,Fe,Co,Ni	hydrothermal	2025	[93]
FeCoCrMnZn	185	10	49.7	–	Cr,Mn,Fe,Co,Zn	hydrothermal	2025	[55]

In the table, *η* represents the overpotential (mV) versus RHE, *j* denotes the current density (mA cm^−2^), *b* refers to the Tafel slope (mV dec^−1^), and *C*_*dl*_ stands for the double-layer capacitance (mF cm^−2^). The tests were conducted under 1 M KOH conditions

While HELHs are predominantly investigated for their exceptional performance in alkaline OER, their stability and potential in acidic media remain a critical area for future exploration. The primary challenge stems from the inherent instability of traditional layered hydroxides in low pH environments, which typically leads to structural degradation [96]. However, recent studies on related high-entropy oxide (HEO) materials have demonstrated promising strategies to overcome these limitations. For instance, constructing high-entropy oxides has been shown to suppress lattice oxygen diffusion, thereby improving stability in acidic OER [97]. The inherent lattice disorder within HEOs increases the migration energy barrier for lattice oxygen, a key factor in the degradation of catalysts like RuO_2_ in acidic environments [98]. Furthermore, a new class of acid-stable HEO nanoparticles with a specific composition was recently synthesized, showcasing superior long-term stability of 12 h in 0.5 M H_2_SO_4_ electrolyte and an overpotential of 370 mV at 10 mA cm⁻^2^ compared to commercial RuO_2_ catalysts [99]. These findings suggest that the same principles of high-entropy stabilization that protect HEOs could be applied to HELHs. The inherent thermodynamic stability and lattice disorder of high-entropy systems increase the energy barrier for degradation. By strategically leveraging cationic synergy and local structural distortions, it may be possible to engineer HELHs with enhanced resistance to acid corrosion. While challenging, designing acid-stable high-entropy materials is a viable and important research direction that could expand their applications beyond alkaline environments. Future work should focus on leveraging these shared features to develop acid-stable HELHs and gain a deeper understanding of their degradation mechanisms in acidic media.

Likewise, HELHs face potential degradation mechanisms under harsh operating conditions. A primary challenge is metal ion dissolution, where some metal components can leach out of the catalyst lattice into the electrolyte over extended operation, leading to a loss of active sites. Catalysts can also undergo structural reconstruction under strongly oxidizing potentials. This dynamic process may cause the layered structure to partially transform into amorphous or crystalline oxides/hydroxides, which can eventually lead to structural collapse and performance decay. These degradation pathways are often intensified by extreme operating conditions. In particularly, high current densities can cause the accumulation of oxygen bubbles on the catalyst surface. This not only blocks active sites but can also lead to localized overheating and severe fluctuations in pH, which may accelerate chemical dissolution and structural collapse. Industrial OER processes typically operate at higher temperatures and pressures, which can accelerate chemical dissolution or structural damage.

Another significant challenge is industrial scalability. While laboratory tests often use small electrodes, the much larger surface area of industrial electrodes can lead to non-uniform current and heat distribution, accelerating localized degradation. To ensure the reliability of stability assessments, it is crucial to use standardized testing protocols that specify clear operating conditions. Advanced characterization techniques are required to be employed to analyze changes in the surface and chemical state of the catalyst before and after testing. Although the high-entropy effect, through multi-element synergy and lattice distortion, helps suppress phase segregation and improve thermodynamic stability, completely eliminating these failure mechanisms remains a challenge.

In summary, HELHs have emerged as a versatile platform for electrocatalytic water oxidation, demonstrating exceptional performance through strategies like entropy stabilization, lattice distortion, and multi-metal electronic coupling. Crucially, the multi-metallic synergy within HELHs offers an unprecedented opportunity to precisely modulate OER reaction mechanisms. By systematically tuning their composition, we can influence the reaction pathway to favor either the conventional AEM or the kinetically superior LOM. For instance, specific elemental combinations can activate the LOM, allowing lattice oxygen to directly participate in O–O bond formation, thereby bypassing the activity limitations of the AEM. This ability to regulate the LOM is key to explaining the exceptionally high OER activity observed in certain HELH systems. Their application in seawater electrolysis and urea-assisted water electrolysis further broadens their potential impact. However, challenges remain in optimizing synthesis methods for industrial scalability, enhancing long-term durability, and, most importantly, achieving a deeper understanding of the underlying catalytic mechanisms. Looking ahead, a more profound understanding of the structure–property relationships in HELHs will be essential for developing next-generation catalysts that are not only highly active but also stable and scalable.

### Hydrogen Evolution and Glucose Electrooxidation: Synergy of Multi-Element Active Sites

HELHs have attracted significant attention in energy conversion catalysis. While extensive studies have been conducted on their application in OER, recent research has highlighted their potential advantages in hydrogen evolution reaction (HER) and glucose electrooxidation reaction (GOR). In HER, HELHs materials exhibit promising activity and durability by leveraging built-in electric fields and multi-metal interactions to optimize hydrogen adsorption and desorption. In GOR, defect-rich HELH catalysts have demonstrated outstanding efficiency in glucose-to-glucaric acid conversion through multi-site synergistic catalysis. These advancements underscore the versatility of HELH materials and their potential for broader applications in electrocatalysis beyond OER.

Green strategies for the sustainable utilization of industrial waste have been developed to address global environmental challenges. Among these challenges, the search for clean and efficient energy sources remains a top priority. Hydrogen is the most ideal clean energy source, offering a promising alternative to fossil fuels due to its high energy density and zero-carbon emissions.

**Fig. 8:**
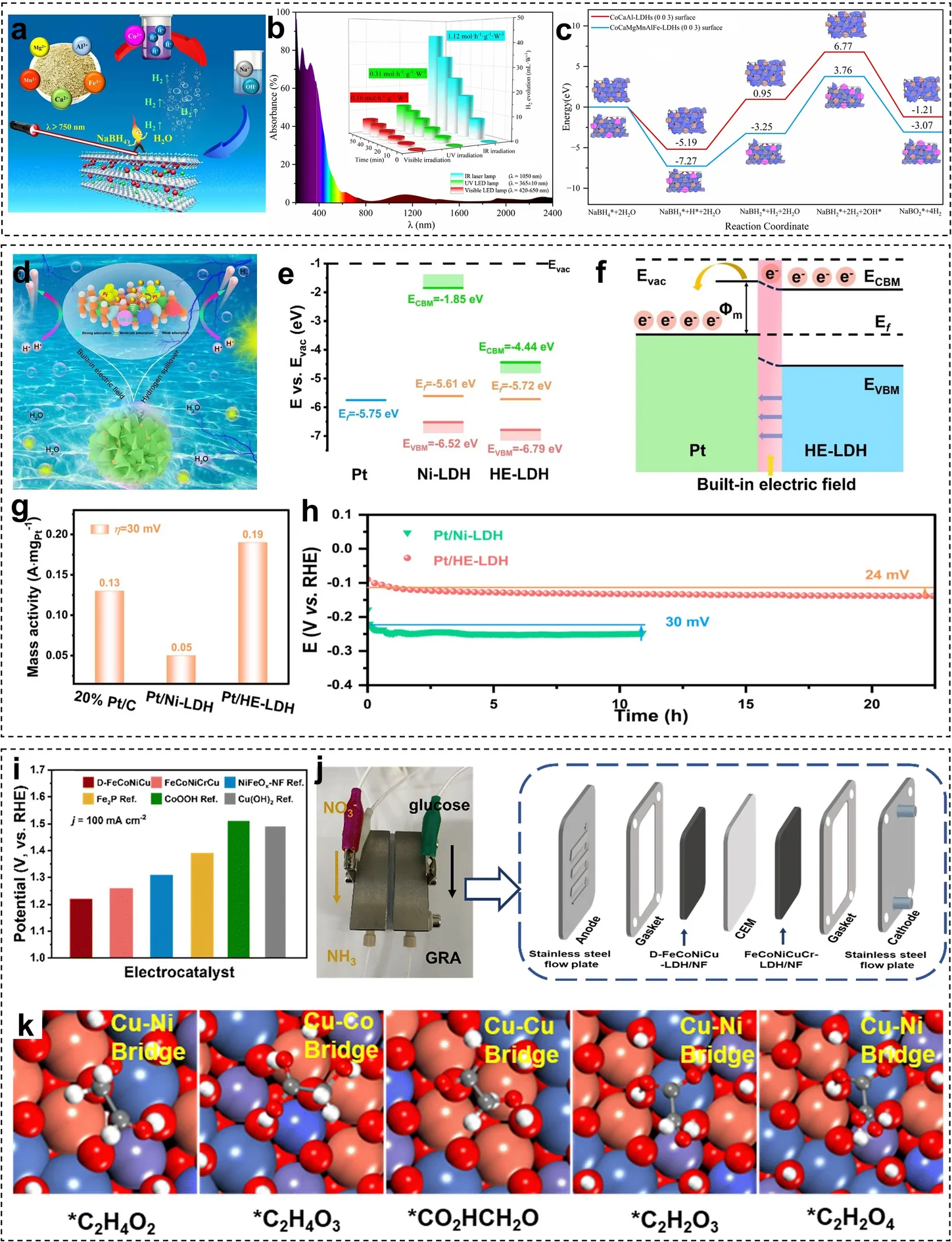
Multifunctional HELHs for hydrogen evolution, glycerol oxidation. **a** Schematic illustration of CoCaMgAlFeMn-LDHs enabling controlled hydrogen generation via NaBH_4_ hydrolysis. **b** UV–Vis–NIR diffuse reflectance spectrum of CoCaMgMnAlFe-LDHs, with the inset showing hydrogen generation rates under different light sources at 298 K. **c** DFT-simulated energy profiles for catalytic NaBH_4_ hydrolysis on low-entropy CoCaAl-LDHs (003) and high-entropy CoCaMgMnAlFe-LDHs (003). Reproduced with permission [100] Copyright 2024, Elsevier. **d** Schematic of Pt nanoparticle-modified NiFeCoCuCr high-entropy layered double hydroxides (Pt/HELHs) with a strong built-in electric field (BIEF) for enhanced acidic hydrogen evolution reaction. **e** Energy band diagram of Pt, Ni-LDH, and HELHs. **f** Schematic representation of BIEF-driven electron transfer at the Pt/HELHs interface. **g** Mass activity comparison of 20% Pt/C, Pt/Ni-LDH, and Pt/HELHs at an overpotential of 30 mV. **h** Chronopotentiometric (CP) curves of Pt/HELHs and Pt/Ni-LDH at 10 mA cm^−2^. Reproduced with permission [102] Copyright 2025, Elsevier. **i** Comparison between D-FeCoNiCu-LDH/NF, FeCoNiCuCr-LDH/NF, and reported electrocatalysts for the GOR in potential required for η_j=100_. **j** Photograph and schematic for the flow electrolyzer for the GOR at the anode and NO_3_ -RR at the cathode. **k** Schematic for active adsorption sites for differing reactants. Reproduced with permission [54] Copyright 2024, Royal Society of Chemistry

Building on this, a method for synthesizing high-entropy CoCaMgMnAlFe-LDH catalysts from steel slag and lithium-ion battery waste has been reported [100]. Metal cations from steel slag (Ca^2+^, Mg^2+^, Al^3+^, Fe^3+^, Mn^2+^) were recovered using acidic and alkaline waste solutions containing Co^2+^, H⁺, NO_3_^−^, Li⁺, Na⁺, OH⁻, and CO_3_^2−^. The catalyst was found to facilitate controllable hydrogen generation via NaBH_4_ hydrolysis while ensuring that no hazardous metal residues remained, allowing safe environmental reintegration (Fig. 8a). Furthermore, the presence of Co^2+^ was observed to enhance photothermal conversion, enabling rapid heating under infrared irradiation and achieving a hydrogen generation rate of 1.12 mol h^−1^ g^−1^ W^−1^ under 1050 nm infrared light. The ultraviolet–visible–near-infrared diffuse reflectance spectrum of CoCaMgMnAlFe-LDHs (Fig. 8b) demonstrates significant light absorption not only in the ultraviolet region (λ = 200–380 nm) but also prominently in the visible range (λ = 380–750 nm). This suggests that Co^2+^ cations effectively absorb near-infrared light, while Fe^3+^ and Mn^2+^ cations efficiently capture visible light. Furthermore, the adsorption energy of high-entropy CoCaMgMnAlFe-LDHs in each elementary reaction is more favorable compared to low-entropy CoCaAl-LDHs, enabling the CoCaMgMnAlFe-LDHs catalyst to efficiently capture a higher quantity of reactive species (Fig. 8c). Similarly, high-entropy NiCoMnAlFe-LDHs have been synthesized from spent LiNi_1-x-y_Mn_x_Co_y_O_2_ cathodes and liquid waste streams, effectively utilizing the residual hazardous metal cations [101]. The HELHs serve as infrared-responsive photothermal catalysts for controllable hydrogen generation via NaBH_4_ hydrolysis, exhibiting excellent HER performance.

Platinum-based catalysts exhibit high HER activity but suffer from instability in acidic environments due to hydrogen buildup and poor durability. To overcome these challenges, Pt nanoparticle-modified NiFeCoCuCr high-entropy LDHs (Pt/HELHs) have been developed by Xu et al. [102], offering enhanced performance and stability. A built-in electric field (BIEF) at the Pt/HELHs interface facilitates charge redistribution, mitigating the strong hydrogen binding energy that typically hinders desorption (Fig. 8d). When platinum (Pt) contacts a semiconductor, the large work function difference (ΔΦ) facilitates the formation of a Schottky junction, driving charge transfer from high to low levels until equilibrium is reached (Fig. 8e). This results in electron flow from the LDHs to the Pt sites, leaving behind the positively charged donor ions (Fig. 8f). The effective electron transfer from LDHs to Pt enhances the hydrogen adsorption process, significantly improving catalytic efficiency. The Pt/HELHs catalyst achieve a remarkable mass activity of 0.19 A mgPt^−1^ at an overpotential of 30 mV, far exceeding that of commercial Pt/C and three times greater than Pt/Ni-LDH (Fig. 8g). Furthermore, the Pt/HELHs show a mere 24 mV increase in overpotential, further demonstrating its excellent electrochemical stability (Fig. 8h).

In addition, the GOR offers a sustainable method for producing high-value chemicals like glucaric acid. To develop efficient catalysts, Wu et al. [54] introduced defect-rich high-entropy LDH nanosheets on nickel foam as a highly active and stable GOR catalyst. The synthesized FeCoNiCu high-entropy LDH achieved a low potential of 1.22 V versus RHE at 100 mA cm^−2^ (Fig. 8i), with near-total glucose conversion and glucaric acid yield over 90%. Additionally, coupling this catalyst with nitrate reduction (NO_3_-RR) in a hybrid flow electrolytic cell (Fig. 8j) demonstrated energy-efficient operation, requiring only 1.07 V at 10 mA cm^−2^ and 1.32 V at 100 mA cm^−2^. Mechanistic studies revealed that multi-site catalytic synergy drives the reaction, with Cu–Co bridges promoting hydroxyl dehydrogenation, Cu–Cu bridges enhancing aldehyde formation, and Cu–Ni bridges accelerating aldehyde oxidation (Fig. 8k).

Recent efforts have also focused on alternative anodic reactions to reduce overall energy consumption, and electrocatalytic methanol oxidation reaction (MOR) has been recognized as an effective alternative to the OER, primarily due to the significantly high overpotential associated with OER, which leads to excessive energy consumption. Wang et al. [103] synthesized a ZnNiFeCoV-HELH that exhibited excellent stability, with the formate yield remaining above 95% even after five cycles relative to the initial performance. The incorporation of vanadium (V), which possesses a relatively large tensile strain compared to Cr and Al, was found to optimize the d-band center of V-HELH, thereby effectively reducing the energy barrier. The tunable electronic structure of HELHs endows them with remarkable versatility across various electrocatalytic reactions, underscoring their promising future in diverse energy-related fields.

HELH materials have demonstrated significant potential in HER and GOR, with experimental validation confirming their excellent catalytic activity and stability. However, beyond HER and GOR, the application of HELHs in other electrochemical conversion reactions, such as nitrogen reduction reaction (NRR), CO_2_ reduction reaction (CO_2_RR), and alcohol oxidation reactions, remains largely unexplored. The broader potential of HELHs in other electrocatalytic conversion processes represents a promising direction for future research, necessitating further investigation to fully elucidate their catalytic mechanisms, optimize structural designs, and expand their practical applications.

Overall, based on an in-depth analysis of HELHs in OER, HER, and GOR, these materials exhibit several key advantages. First, their high-entropy effect, through the synergistic interplay of multiple metal components, enables the fine-tuning of electronic structures and the optimization of adsorption energies for reaction intermediates. This significantly lowers reaction barriers and enhances catalytic activity. Second, lattice distortion induced by the coexistence of multiple metals and a unique multi-site synergistic mechanism not only accelerates reaction kinetics but also effectively enhances structural stability, ensuring long-term durability. Furthermore, flexible structural designs, such as the introduction of vacancies, defects, or heterostructures, offer ample opportunities for further performance optimization. Essentially, with their unique compositional, structural, and electronic properties, HELHs has emerged as a powerful platform for designing highly efficient electrocatalysts.

## HELHs for Advanced Energy Storage and Biomedical Innovations

HELHs represent transformative materials that integrate energy storage and biomedical applications. These materials exhibit nanostructured architectures characterized by exceptional ionic conductivity, stable cycling performance, and entropy-enhanced thermodynamic resilience. HELHs demonstrate superior performance as electrocatalysts in critical energy reactions, such as oxygen evolution and hydrogen evolution, while enabling precise tumor therapy through tunable bandgap engineering. HELHs-based systems hold significant potential for photocatalytic activation, synergistic antitumor therapy, and sensitive antibiotic detection. High-entropy effects enhance catalytic activity, reactive oxygen species (ROS) generation, tumor microenvironment reprogramming, and peroxidase-mimicking capabilities for tetracycline sensing. Future research directions emphasize the integration of machine learning algorithms for compositional optimization and the development of scalable synthesis methods to facilitate practical applications.

### High-Entropy Engineered Materials for Batteries and Supercapacitors: Toward Efficient and Stable Energy Storage

HELHs have emerged as promising materials for energy storage devices such as batteries and supercapacitors due to their unique multi-component synergy and tunable electronic structures. Despite their potential, applications of HELHs in these fields remain underexplored, primarily due to synthesis challenges, limited conductivity. Addressing these limitations is crucial to unlocking their full potential in advanced energy storage systems.

**Fig. 9:**
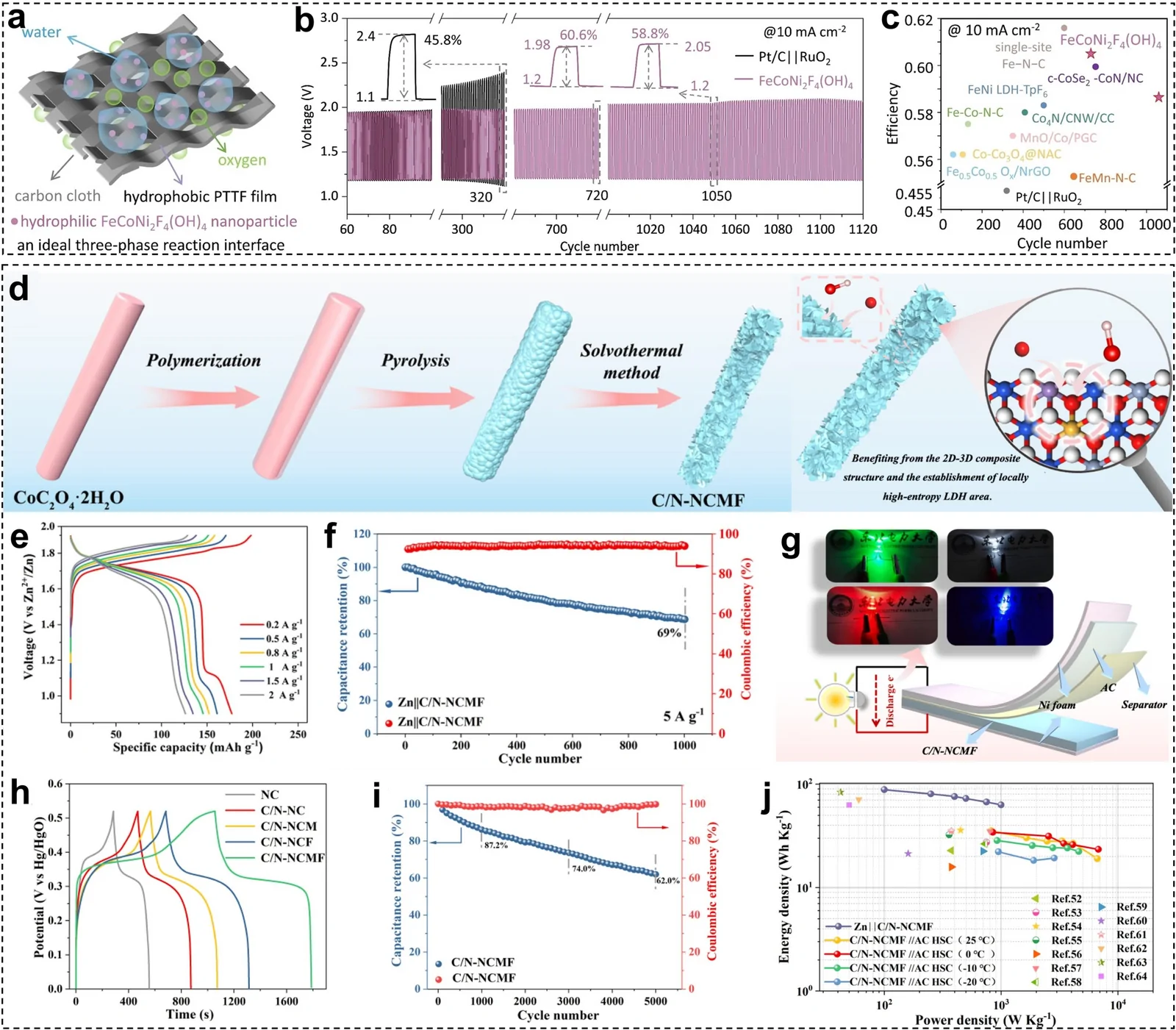
HELHs for advanced batteries and supercapacitors. **a** Schematic illustration of an ideal three-phase reaction interface for rapid O_2_ transport in zinc-air batteries (ZABs) using FeCoNi_2_F_4_(OH)_4_@HCC and Pt/C||RuO_2_@HCC cathodes. **b** Round-trip energy efficiency (discharge-to-charge voltage ratio) and long-term durability at 10 mA·cm^−2^, with an inset showing detailed charge–discharge curves for selected cycles. **c** Comparison of energy efficiency and stability for ZABs employing FeCoNi_2_F_4_(OH)_4_@HCC cathodes versus previously reported ZABs at 10 mA cm^−2^. Reproduced with permission [104] Copyright 2024, Wiley. **d** Schematic representation of C/N-NCMF preparation and its charge storage mechanism. **e** Galvanostatic charge–discharge (GCD) curves of the Zn||C/N-NCMF battery at various current densities. **f** Cycling performance profile of the Zn||C/N-NCMF battery. **g** Conceptual schematic of the C/N-NCMF-based hybrid supercapacitor. **h** Comparative GCD profiles of electrodes using the as-synthesized materials. **i** Cycling performance of C/N-NCMF electrodes. **j** Ragone plot showing energy and power densities for Zn batteries and hybrid supercapacitors. Reproduced with permission [105] Copyright 2024, Elsevier

Li et al. [104] introduced a fluorine-doped high-valence HELHs (FeCoNi_2_F_4_(OH)_4_) synthesized via multi-ion co-precipitation. The F^−^ ions were firmly embedded within individual hydroxide layers. Spectroscopic analysis and theoretical simulations revealed the presence of high-valence metal cations, which expanded the metal 3d and O 2p orbital overlap, thereby improving electronic conductivity and OER catalytic activity. An efficient three-phase (gas/liquid electrolyte/solid catalyst) reaction interface with fast O_2_ diffusion was constructed to accelerate the sluggish ORR (Fig. 9a). Additionally, the robust M–O(F)_6_ octahedral geometry prevented structural degradation, enabling long-term stability. The resulting zinc-air batteries (ZAB), employing FeCoNi_2_F_4_(OH)_4_@HCC cathodes, exhibited superior round-trip efficiency (61.3% at 10 mA·cm^−2^) and retained 58.8% efficiency after 1050 cycles, highlighting the material’s potential for realizing metal-air batteries with superior energy efficiency and long-term durability (Fig. 9b, c).

High-performance electrodes are critical for enabling rechargeable devices to operate under extreme conditions, such as high or low temperatures. To enhance zinc battery and supercapacitor performance, C/N co-doped NiCoMnFe-LDH (C/N-NCMF) was engineered by Guan et al. [105] to exploit local high-entropy effects, enhancing electronic structure and conductivity. The synthesis involved three key stages: polymerization, pyrolysis, and solvothermal reaction (Fig. 9d). First, dopamine polymerization, aided by Fe^3+^ and Mn^2+^ ions, produced polydopamine, uniformly embedding metal ions. Pyrolysis compacted the nanorod structures, enhancing composition blending for uniform C/N co-doped LDH composites. Finally, a solvothermal reaction in an ethanol–water mixture hydrolyzed nickel nitrate, releasing OH^−^ and protons. The calcined product, partially etched by protons, released metal ions, which oxidized and co-precipitated with OH^−^ to form high-entropy 2D-3D LDH nanostructures. The porous structure formed by the assembly of nanosheets offers a large contact area with the electrolyte, while the characteristic layered arrangement ensures the abundant exposure of active sites on the surface.

Subsequently, the potential applications of the C/N-NCMF cathode in aqueous rechargeable devices, Zn batteries, and hybrid supercapacitors (HSC) were evaluated. Galvanostatic charge–discharge (GCD) curves indicate a typical battery-type storage process (Fig. 9e). Specific capacities at 0.2–2.0 A g^−1^ ranged from 177 to 126 mAh g^−1^, highlighting the Zn battery’s excellent performance. After 1000 cycles (Fig. 9f), 69% capacity retention and nearly 100% Coulombic efficiency confirm excellent stability. Figure 9g illustrates the HSC schematic, tested by powering an LED. Figure 9h compares GCDs of as-synthesized materials, with C/N-NCMF exhibiting the longest discharging time, emphasizing the strong synergistic effects of its multiple components on electrochemical activity. After 1000 cycles, C/N-NCMF retained 87.2% of its capacitance, and 62% after 5000 cycles (Fig. 9i). Ragone plot (Fig. 9j) accentuates the Zn battery’s higher energy density (88.6 Wh kg^−1^ at 100 W kg^−1^), while the hybrid supercapacitor maintained stable performance across temperatures, showing strong performance.

LDHs have emerged as promising electrode materials for supercapacitors due to their inherently large theoretical specific surface area, which supports electric double-layer capacitance, and the incorporation of transition metal elements within the layers, which serve as active sites for electrochemical reactions, thereby enhancing pseudocapacitance. However, LDHs are constrained by sluggish kinetics in surface-dependent redox reactions and the formation of unstable phase structures, resulting in inherently low energy density [106].

**Fig. 10:**
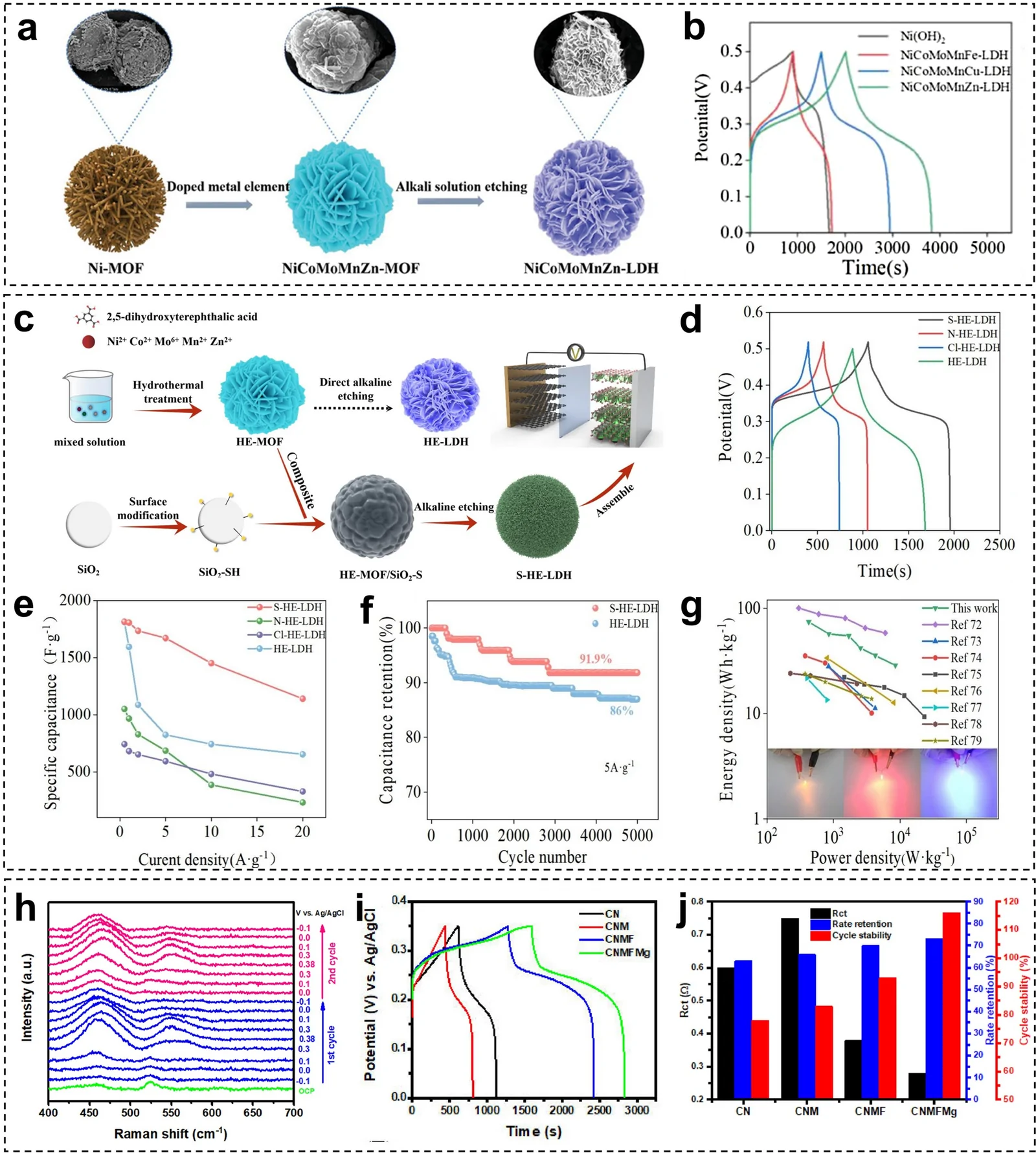
Electrochemical performance of HELHs and their application in hybrid supercapacitors. **a** Schematic representation of the preparation process for NiCoMoMnZn-LDH. **b** GCD curves for Ni(OH)_2_ and HELHs. Reproduced with permission [111] Copyright 2023, Wiley. **c** Synthesis procedure for S-NiCoMoMnZn-LDH (S-HELHs). **d** GCD curves of three HELHs variants doped with different atoms compared to the original HELHs. **e** Specific capacitance vs. current density for four LDH materials. **f** Cyclic performance of S-HELHs electrode material at a current density of 5 A·g^−1^. **g** Ragone plot for S-HELHs//AC HSC, with an additional diagram illustrating application testing. Reproduced with permission [107] Copyright 2024, Elsevier. **h** In situ Raman spectra of the high-entropy hydroxide grown on Ni foam (CNMFMg) recorded at 2 mV s^−1^. **i** GCD curves of hydroxides at 2 mA cm^−2^. **j** The correlation of R_ct_, and rate and specific capacity retentions of hydroxides. Reproduced with permission [110] Copyright 2023, Elsevier

To improve energy density and stability, a five-element NiCoMoMnZn-LDH was synthesized using a novel template re-etching method that exploits configurational entropy (Fig. 10a) [111]. This approach resulted in a material with a uniform elemental distribution, high specific surface area, and a porous structure, which collectively contribute to its exceptional electrochemical performance. The universal synthesis strategy proposed effectively prevents phase separation and elemental aggregation, while minimizing structural distortions, thereby potentially enabling the scalable production of high-entropy hydroxides. GCD tests demonstrated that high-entropy LDH materials significantly outperform single-component Ni(OH)_2_, achieving an impressive specific capacitance of 1810.2 F g^−1^ at a current density of 0.5 A g^−1^ (Fig. 10b). Moreover, a hybrid supercapacitor device assembled with NiCoMoMnZn-LDH achieved an energy density of 62.1 Wh kg^−1^ at a power density of 475 W kg^−1^. Furthermore, DFT calculations provided deeper insights into the synergistic contributions of the different metal components to both structural stability and electrochemical performance.

However, the electrochemical activity of HELHs as electrode materials is still limited by the availability of active sites and their relatively low conductivity, which negatively impacts both specific capacitance and rate performance. To further optimize the microstructure and electronic structure, a novel high-entropy LDH with an S-doped hollow nanoflower shape (S-HELH) was synthesized using a dual-template method combined with nanostructuring techniques (Fig. 10c), aimed at enhancing the energy storage capability and rate performance of the electrode material [107]. Electrochemical performance tests revealed that, at a current density of 1 A g^−1^, the GCD curves showed a significant increase in the electrochemical capacity of S-HELHs (1593.7 F g^−1^) compared to HELHs, which is mainly attributed to the unique structure of S-HELHs (Fig. 10d). Figure 10e compares the specific capacitance of S-HELHs and HELHs at different current densities. As the current density increased, the specific capacitance of HELHs showed a significant drop. However, due to the presence of sulfur ions in S-HELHs, which serve as active sites for redox reactions and accelerate ion transport, S-HELHs maintained a high specific capacitance even at higher current densities. Additionally, after 5000 charge–discharge cycles at a current density of 5 A g^−1^, S-HELHs retained 91.9% of its initial capacitance (Fig. 10f), which is significantly better than HELHs (86.0%). This improvement is mainly attributed to the hollow structure of S-HELHs, which effectively suppresses structural deformation and layer accumulation during the charge–discharge process. Finally, the S-HELHs/AC hybrid supercapacitor achieves an impressive energy density of 74.2 W kg^−1^ at a power density of 475 W kg^−1^ (Fig. 10g). Compared to other reported hybrid supercapacitors, this device exhibits both higher energy and power densities, further validating the effectiveness of combining high-entropy strategies with structural optimization in enhancing electrochemical performance.

To enhance the energy density and cycling stability of supercapacitors, phase transition behavior and charge transfer dynamics are critical factors to consider [108, 109]. Regarding phase stability, in situ Raman spectroscopy (Fig. 10h) reveals that the reversible phase transition of Mn, Fe, and Mg doped NiCo hydroxide (CNMFMg) during charge/discharge cycling can be effectively regulated [110]. This structural modulation results in superior cycling stability for CNMFMg. Additionally, the high-entropy strategy not only enhances the specific capacitance, reaching up to 2476 mC cm^−2^ at 2 mA cm^−2^ (Fig. 10i), but also improves rate capability, maintaining 73% of its capacitance at 10 mA cm^−2^, along with excellent cycling stability, where the capacitance increases to 116% of its initial value after 2000 cycles.

For charge transfer resistance analysis (Fig. 10j), a lower charge transfer resistance corresponds to improved rate retention. Thus, the remarkable rate capability and cycling stability of CNMFMg can be attributed to its high electrical conductivity, which facilitates efficient charge transport.

In brief, HELH materials, through the integration of high-entropy strategies, microstructural optimization, and electronic structure regulation, have shown exceptional performance in energy storage and conversion applications such as supercapacitors and OER. These advancements provide valuable insights for the efficient energy storage and long-term stable operation of future energy storage devices. However, despite their promising potential, the widespread application of HELHs is still hindered by challenges in synthesis and interface control. Therefore, future research should prioritize refining fabrication techniques, exploring the multi-component synergies in more depth, and enhancing interfacial properties to enable the broader adoption of HELHs in next-generation energy storage technologies and catalytic applications.

### Photocatalytic ROS Generation and Immune Microenvironment Remodeling for Synergistic Antitumor Therapy

Recent application studies on HELH-based systems for photocatalytic activation and immune synergistic antitumor therapy underscore the versatile potential of these materials. Their unique high-entropy effects fine-tune active site modulation and electronic configurations, driving efficient catalysis and robust bioactivity not only in battery and capacitor systems but also in advanced photocatalytic and biomedical applications.

**Fig. 11:**
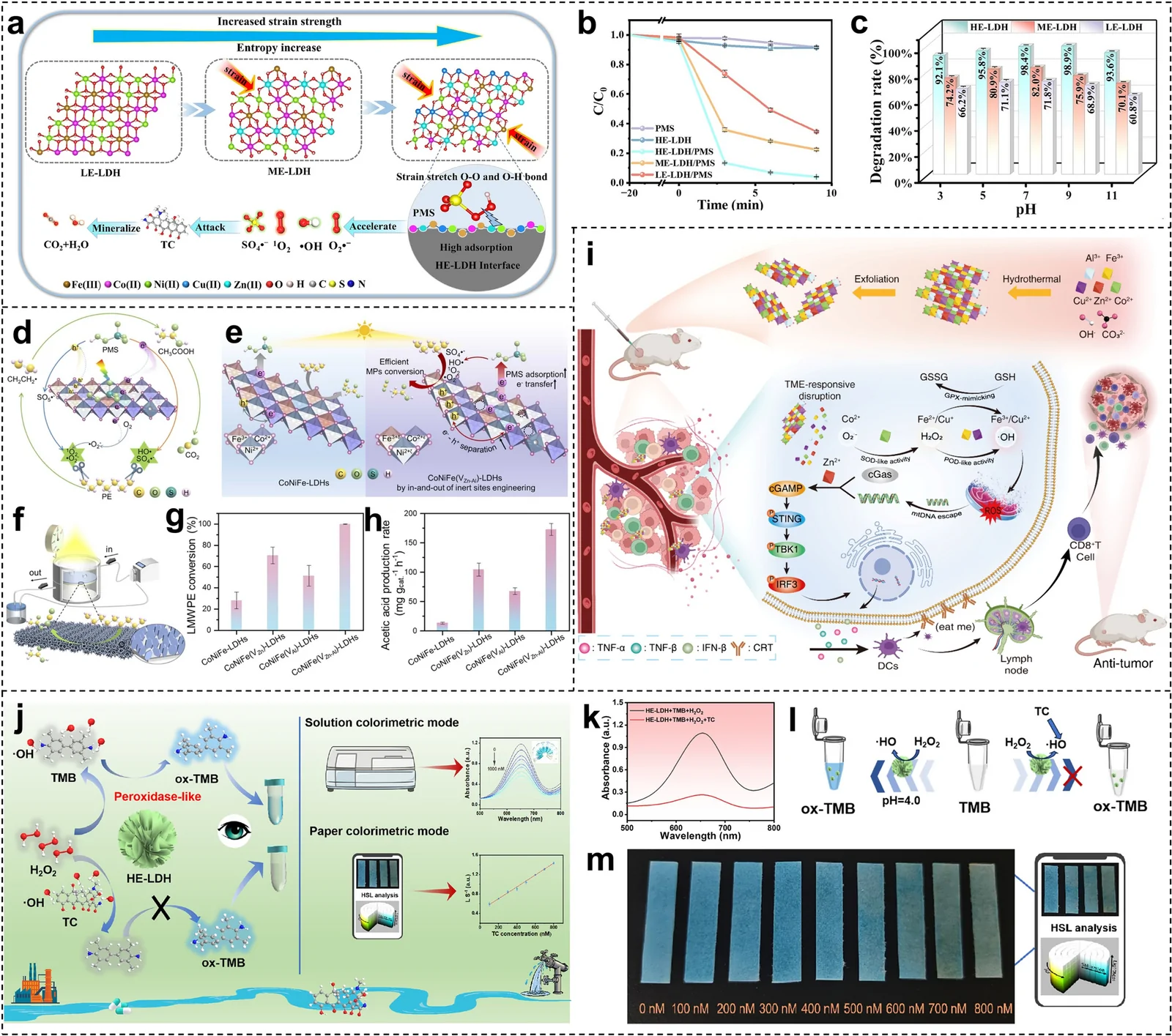
HELH-based systems for photocatalysis and synergistic immunotherapy in antitumor applications. **a** Mechanistic analysis of entropy-driven strain effects in HELHs for enhanced peroxymonosulfate (PMS) activation. **b** Tetracycline (TC) degradation curves for various catalytic systems. **c** Comparative performance of different systems under identical pH conditions. Reproduced with permission [112] Copyright 2025, Elsevier. **d** Proposed pathways for active species generation and polyethylene (PE) molecule decomposition in the PMS-assisted photocatalytic system. **e** Scheme of constructing high-valence metal sites on CoNiFe(V_Zn-Al_)-LDHs for improved microplastics (MPs) conversion. **f** Schematic diagram of the developed flow photocatalytic system. **g** Low molecular weight PE (LMWPE) conversion and **h** the corresponding acetic acid production rates in PMS-assisted photocatalytic systems across different samples. Reproduced with permission [113] Copyright 2025, Elsevier. **i** Schematic representation of the HELHs synthesis process and its antitumor strategy of nanozyme-initiated chemodynamic therapy (NCDT) and immune synergistic therapy. Reproduced with permission [114] Copyright 2024, American Chemical Society. **j** Schematic Diagram of Dual-Mode Colorimetric Sensor for TC Detection Based on the HELHs Peroxidase-Mimic Nanozyme. **k** Absorption spectra of HELHs + TMB + H_2_O_2_ and HELHs + TMB + H_2_O_2_ + TC. **l** Mechanism of colorimetric detection of TC based on HELHs. **m** Pictures are shown after the reaction of various concentrations TC and HELHs on the test paper. Reproduced with permission [115] Copyright 2025, American Chemical Society

For instance, it has been demonstrated that the high-entropy effect induces a pronounced strain within the FeCoNiCuZn-LDH structure [112], effectively shortening metal–oxygen–hydrogen (M–O–H) bond lengths and enhancing stability. In comparison with FeCoNiZn-LDH (ME-LDH) and FeCoNi-LDH (LE-LDH), the elevated strain in high-entropy LDH (HELH) effectively modulates the geometry of the reaction interface (Fig. 11a). This modulation not only increases the adsorption energy at the active sites but also elongates the O–O and O–H bonds in peroxymonosulfate (PMS). Consequently, this structural adjustment significantly promotes the generation of reactive oxygen species for efficient PMS activation and facilitates rapid, robust degradation of pollutants such as tetracycline (TC). Remarkably, the HELHs/PMS system achieved approximately 90% degradation within 3 min of PMS addition and nearly complete degradation within 10 min (Fig. 11b), whereas the ME-LDH/PMS and LE-LDH/PMS systems only achieved removal rates of 43.2% and 27.4%, respectively. Furthermore, the HELH/PMS system exhibited optimal performance over an initial pH range of 5–9, with degradation efficiencies exceeding 95% (Fig. 11c). These findings underscore the efficacy of HELHs in activating PMS and its ability to remove tetracycline across a broad pH range (pH = 3–11), highlighting its substantial potential for practical applications.

To facilitate peroxymonosulfate-assisted photocatalytic removal of microplastics (MPs), Wu et al. [113] precisely manipulated inert Zn/Al sites within CoNiFe(V_Zn-Al_)-LDHs to optimize the electronic configuration of metal sites. The pathways of active species generation and the polyethylene (PE) decomposition in the PMS-assisted photocatalytic system are proposed (Fig. 11d). By dynamically modulating these inert sites, high-valence transition metal centers are formed on CoNiFe(V_Zn-Al_)-LDHs. Consequently, the modified bandgap of these LDHs affords enhanced light-harvesting capabilities and more efficient electron–hole separation relative to CoNiFe-LDHs. Moreover, the generated Fe^3+δ^, Co^2+ε^, and Ni^2+ζ^ sites promote the activation of PMS, leading to the production of abundant reactive species. As a result, CoNiFe(V_Zn-Al_)-LDHs exhibit excellent performance in removing MPs (Fig. 11e). The performance of the PMS-assisted photocatalytic system was evaluated using a custom-built stainless steel flow reactor under full-spectrum irradiation (λ > 200 nm) (Fig. 11f). Compared with all alkali-etched LDH samples, the NF@CoNiFe(V_Zn-Al_)-LDHs deliver a significantly improved low molecular weight polyethylene (LMWPE) conversion efficiency (approaching 100%, Fig. 11g). Notably, acetic acid, the primary degradation product, was detected (Fig. 11h), with the NF@CoNiFe(V_Zn-Al_)-LDHs achieving an acetic acid yield rate of 173.1 mg g^−1^ h^−1^, which is 12.8 times higher than that of NF@CoNiFe-LDHs.

High-entropy nanomaterials have garnered significant attention in the fields of energy and catalysis; however, their biomedical applications remain largely unexplored. Wang et al. [114] developed a two-dimensional HELH nanoplatform that achieves efficient antitumor activity by reprogramming the tumor microenvironment (TME) and integrating cascade nanozyme-initiated chemodynamic therapy (NCDT) with immune synergistic therapy (Fig. 11i). HELHs release Co^2+^, Fe^3+^, and Cu^2+^ ions, exhibiting superoxide dismutase (SOD), peroxidase (POD), and glutathione peroxidase (GPX) activities to continuously generate reactive oxygen species (ROS), thereby alleviating tumor hypoxia and enhancing NCDT efficacy. Simultaneously, the released Zn^2+^ ions activate the cyclic GMP-AMP synthase/stimulator of interferon gene (cGAS/STING) signaling pathway, strengthening the innate immune response. In vivo tumor models demonstrated that this strategy significantly upregulated antitumor cytokines and activated T cells, leading to superior therapeutic outcomes. This study not only establishes a novel research direction but also provides a theoretical foundation and technological framework for the integration of high-entropy nanomaterials into advanced healthcare applications for simultaneous NCDT and immunotherapy.

Monitoring antibiotic residues is essential for mitigating bacterial resistance and protecting environmental and human health. Among various detection techniques, sensor-based approaches have attracted significant attention due to their potential for rapid, sensitive, and portable analysis. However, it remains challenging to integrate high sensitivity, selectivity, and portability into a single sensor platform for the effective detection of tetracycline (TC). A flower-like spherical high-entropy layered double hydroxide (Fe_0.45_Co_0.45_Ni_0.45_Cu_0.45_Zn_0.45_-LDH, HELHs) was synthesized and exhibited excellent peroxidase-like activity, efficiently converting H_2_O_2_ into hydroxyl radicals (∙OH) [115]. This catalytic performance was mainly attributed to the multivalent metal centers (Fe, Co, Ni). Based on the high reactivity between TC and the generated ∙OH, a simple and effective colorimetric sensing strategy for tetracycline (TC) detection was developed (Fig. 11j). TC competes with 3,3′,5,5′-tetramethylbenzidine (TMB) for ∙OH, thereby inhibiting the oxidation of TMB and reducing the expected colorimetric response.

With the addition of TC, ∙HELHs exhibited poor catalytic properties through the complexation of TC, which inhibited the oxidation of TMB and consequently reduced the absorbance (Fig. 11k). HELHs were used as a catalyst to oxidize TMB to blue ox-TMB in the presence of ∙OH by using its POD-like property, while the addition of TC could compete with TMB to generate ∙OH in the catalytic reaction, thus inhibiting the color reaction of TMB (Fig. 11l). According to the different degrees of discoloration and the molar extinction law, the relationship between the absorbance and the concentration of TC detected could be established. Thereby, a colorimetric sensor based on HELHs can be constructed for sensitive detection of TC. Consequently, a portable paper-based sensing platform was also established, where a mixture of HELHs, H_2_O_2_, and TMB was deposited on filter paper; upon addition of TC solution, blue-to-light-blue color shifts were observed (Fig. 11m). The test paper exhibited a color change in response to varying TC concentrations, and the hue was quantified through smartphone-based analysis using digital color signals (H, S, and L values), enabling both visual and quantitative detection of TC. The work highlights the potential of HELHs in antibiotic detection and provides a valuable strategy for the design of next-generation point-of-care sensing systems. Future efforts should prioritize the structural engineering and compositional modulation of HELHs to further enhance their environmental adaptability and sensing performance, enabling more precise, stable, and selective detection of antibiotic residues across diverse environmental matrices.

**Fig. 12:**
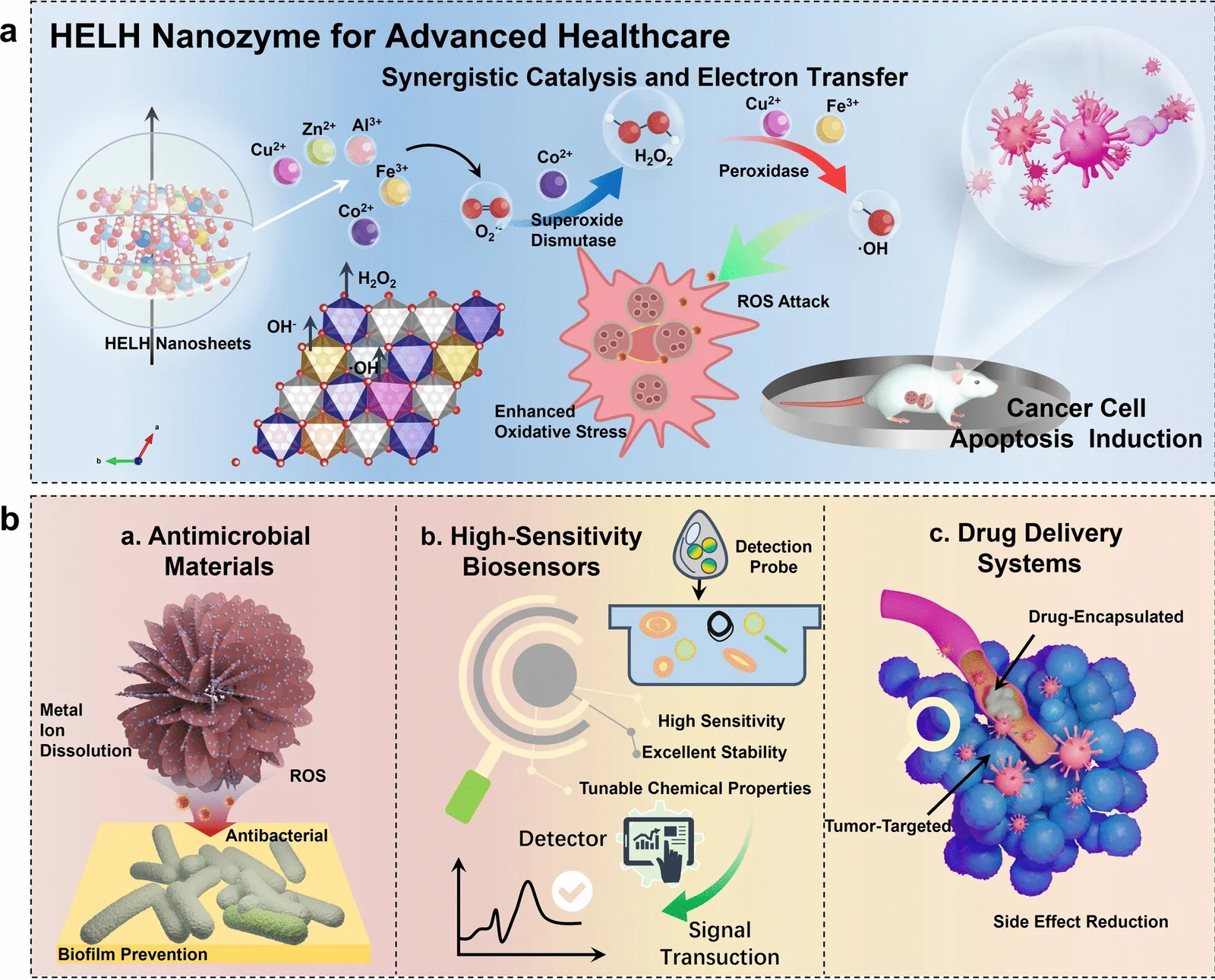
Synergistic catalysis and advanced biomedical applications of HELHs nanozymes. **a** Synergistic catalysis and electron transfer: HELHs act as nanozymes, leveraging multi-metal sites to form an efficient electron transfer chain. This synergistic action allows for the robust generation of reactive oxygen species (ROS) to promote stronger oxidative stress and induce cancer cell apoptosis. **b** Future biomedical applications: The unique properties of HELHs open up new possibilities in various biomedical fields, including the development of advanced antimicrobial materials, high-sensitivity biosensors, and targeted drug delivery systems

Although the precise mechanisms underlying the biological functions of HELHs are not yet fully elucidated, several proposed pathways provide promising directions for future investigation. The unique multi-metal synergy of HELHs provides a new avenue for powerful antitumor activity. As depicted in Fig. 12a, HELHs act as nanozymes, with their multi-metal sites forming an efficient electron transfer chain that significantly boosts ROS generation [114]. In this synergistic process, HELHs are degraded at low pH, allowing them to simulate multiple nanozymes and act as ROS producers by catalyzing the dismutation of O_2_^∙−^ into ∙OH. The release of Co^2+^ endows HELHs with SOD activity. With multiple sites coexisting in HELHs, the material shows a high affinity for H_2_O_2_. The POD activity of HELHs is stronger than that of other reference LDHs. Fe^2+^ can catalyze H_2_O_2_ to generate the highly reactive hydroxyl radical (∙OH). Cu^2+^ can further catalyze to produce ∙OH through a Fenton-like reaction (Cu(I)/Cu(II) cycle). The synergistic effect of these metal sites accelerates the Fenton-like reaction. By acting together, the metal sites more efficiently consume in the tumor microenvironment to produce more ∙OH, leading to stronger oxidative stress and inducing cancer cell apoptosis.

Future research could delve deeper into how to design and control these multi-metal sites to form a precise cascade reaction system, as the catalytic efficiency of such a system may far exceed that of a single metal. In addition to tuning electronic properties, it might be possible to manipulate the geometrical structure to modulate the immune microenvironment. By leveraging their unique surface characteristics, these materials could be designed to interact with biomolecules, thereby enhancing immune activation. While many of the mechanisms for HEMs in biomedical applications remain to be fully explored, their unique properties offer vast potential in various fields, as illustrated in Fig. 12b. Beyond antitumor therapy, HELHs can be used to develop novel antimicrobial materials by utilizing their catalytic activity to disrupt bacterial cell membranes or inhibit metabolic processes. Their high surface area and tunable chemical properties also make them excellent candidates for high-sensitivity biosensors. Furthermore, HELHs show great promise as drug delivery systems, capable of precisely loading drugs and releasing them in a controlled manner within a specific microenvironment. A comprehensive understanding of these materials will require a combination of advanced material analysis and biomedical techniques to fully reveal the mechanisms and potential pathways of HELHs in medicine.

In conclusion, HELHs have demonstrated exceptional electrochemical performance in energy storage devices such as zinc-air batteries and supercapacitors, owing to their unique catalytic activity and multifunctionality. Beyond energy applications, these materials also exhibit significant potential in chemical conversion, environmental remediation, and biomedical fields. Precise modulation of metal composition and electronic structure has been shown to enhance stability, optimize active sites, and refine reaction interfaces, thereby markedly improving catalytic efficiency. Furthermore, the multifunctional nature of HELH materials suggests their applicability in a broader range of fields, including energy conversion, environmental restoration, chemical synthesis, and biomedical devices, fostering interdisciplinary technological advancements. Continued exploration of their structure–property relationships and multi-field coupling effects will provide critical theoretical insights and technical foundations for the development and large-scale implementation of next-generation high-performance composite materials.

## Conclusion and Future Perspectives: Bridging Synthesis-Characterization-Computation Trinity

HELHs constitute a transformative class of materials that leverage entropy-driven stabilization mechanisms to integrate multifunctional capabilities across energy and biomedical domains. Their chemically tunable architectures, combined with structurally disordered yet thermodynamically stable frameworks, exhibit superior electrochemical performance in oxygen evolution reactions, advanced metal-air batteries, and hybrid supercapacitors. These catalytic advancements are particularly compelling when contextualized against the prevailing single-atom (SA) site paradigm. While SA catalysts, including advanced inner-hosted structures, excel at maximizing atomic efficiency, their performance is often limited to a single type of active site [116]. The field has sought to introduce cooperativity through bimetallic SA catalysts (bimSACs) [117]. HELHs, however, represent a different approach to this concept. Instead of 1–2 isolated atoms on a support, HELHs embed 5 or more different cations directly into the host lattice. This creates a high density of multi-metal synergistic sites, leveraging the “cocktail effect” in a manner distinct from simpler SA systems. This multi-element strategy, also proven effective in high-entropy amorphous catalysts [118], offers a specific advantage in HELHs, where the defined layered structure provides a tunable crystalline platform for modulating electronic properties and intermediates, distinguishing them from disordered amorphous phases. Moreover, their versatility extends beyond catalysis, with photon-responsive properties that enable precise tumor therapies through spatiotemporal generation of ROSs. This review highlights the key advancements that make these materials so promising:

1. Innovative Synthesis and Material Design: Innovative synthesis paradigms for HELHs, such as plasma-assisted synthesis and MOF-templated methods, are reviewed, demonstrating great promise through excellent control over morphology and composition. These offer potential pathways to overcome the scalability and cost limitations of traditional synthesis and their use as versatile precursors for other high-entropy materials.
2. Catalytic Superiority and Mechanistic Insights: The review highlights the exceptional electrocatalytic performance of HELHs in OER, HER, and GOR. In-depth mechanistic insights are provided, revealing how multi-metal synergy and entropy-driven effects enable precise modulation of key reaction pathways, such as activating the LOM to bypass conventional scaling limitations.
3. Advanced Applications and Future Directions: This review demonstrates the broad potential of HELHs in advanced applications, including energy storage (e.g., supercapacitors, metal-air batteries) and biomedical fields (e.g., antitumor therapy). We outline critical challenges and future research directions to achieve structural controllability, improve long-term durability, and bridge the gap between laboratory-scale innovation and commercial viability.

Although significant progress has been made in scalable synthesis protocols, critical challenges persist in achieving precise phase control, real-time monitoring of cation/anion redistribution, and accurate computational modeling of entropy-dominated reaction dynamics.

**Fig. 13:**
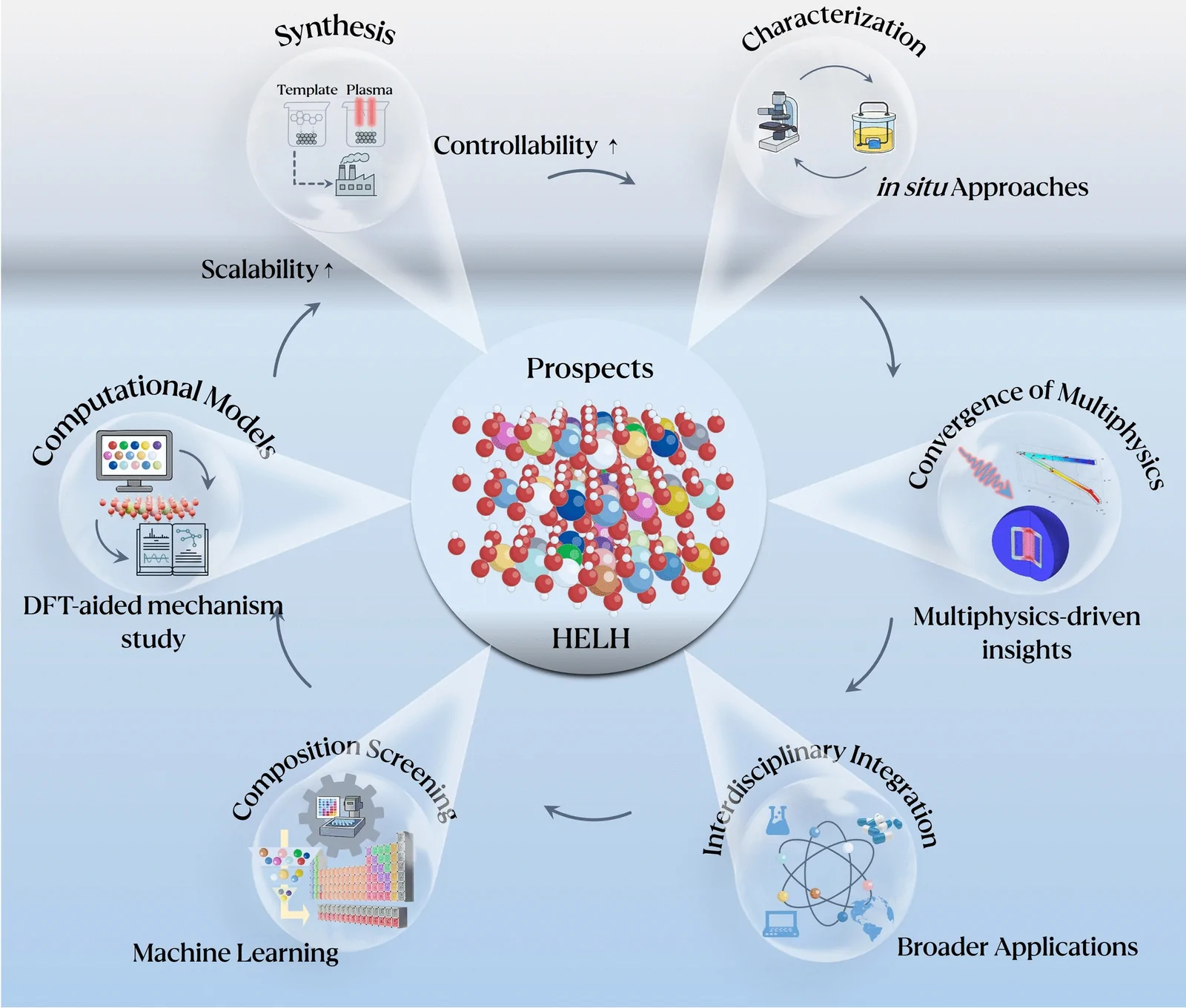
Future Outlook of HELHs Research and Applications. To unlock the full potential of HELHs, future research must focus on enhancing synthetic controllability and scalability, integrating in situ characterization techniques, and merging multiphysics analysis with simulations. Advances in DFT modeling and machine learning-assisted composition screening will further deepen mechanistic understanding and accelerate catalyst optimization. Interdisciplinary collaboration is also essential to expand HELHs applications in catalysis, energy, environment, and biomedicine. These strategies collectively pave the way for rational design and real-world deployment of HELHs materials

Future advancements in HELHs systems necessitate the synergistic integration of operando spectroscopic diagnostics with machine learning-driven combinatorial screening methodologies to elucidate complex interfacial phenomena (Fig. 13). Key innovation pathways encompass: the development of multiscale DFT frameworks incorporating ab initio molecular dynamics simulations and neural network potentials for resolving interfacial charge transfer dynamics; the establishment of cross-disciplinary research consortia merging flexible iontronics, environmental nanozymes, and stimuli-responsive drug delivery architectures. By bridging defect-engineered synthesis protocols with application-oriented material design principles, HELHs are poised to serve as cornerstone materials in next-generation energy storage systems and intelligent medical implants, thereby fundamentally transforming entropy-mediated materials engineering. Persistent challenges in interfacial stability regulation and dynamic structure–property correlations require focused investigation along the following strategic axes:

### Controllability–Scalability Trade-offs in High-Entropy Synthesis

To achieve the uniformity and stability of HELHs, the development of synthesis methods that are efficient, reproducible, and scalable for industrial production represents a critical priority. Future research should concentrate on integrating emerging technologies to refine and optimize established techniques such as hydrothermal synthesis, co-precipitation, plasma etching, structure-directed assembly, and template-assisted etching, with the overarching goal of constructing a highly controllable and versatile synthesis framework. Such efforts are essential not only for deepening the fundamental understanding of HELHs but also for accelerating their translation from laboratory-scale fabrication to real-world applications.

### Operando Spectroscopy and Atomic-Resolution Imaging for Real-Time Process Monitoring

The comprehensive elucidation of the structure–property relationships and catalytic performance of HELHs necessitates the integration of multiple advanced characterization techniques, with a particular focus on in situ methodologies. For instance, in situ scanning electrochemical cell microscopy (SECCM) at nanoscale spatial resolution enables real-time monitoring of active site dynamics on catalytic surfaces during operation, offering mechanistic insights into the correlations between structure and catalytic activity under working conditions. Such multimodal characterization strategies are indispensable for establishing robust structure–property relationships, thereby informing rational catalyst design and facilitating performance optimization.

### Multiphysics-Experimental-Simulation Integration for Multiscale Mechanistic Insights

Investigating the behavior of high-entropy materials under diverse physical fields is of critical significance. The integration of experimental techniques, such as thermogravimetric analysis (TGA) combined with in situ heating transmission electron microscopy (TEM) or optical microscopy, facilitates comprehensive observation of material transformations under thermal conditions. Furthermore, simulation tools like COMSOL can be utilized to model pressure-strain relationships, electrostatic interactions, and static magnetic fields. The synergy between experimental validation and theoretical modeling provides valuable insights, elucidating the catalytic effects of high-entropy transition metals under magnetic influences and enhancing the understanding of their fundamental physical properties. Such a combined experimental-simulation approach offers a robust strategy for unraveling the complex behavior of high-entropy materials across various physical environments.

### DFT-Driven High-Throughput Screening of Entropy-Stabilized Configurations

In light of the complexity of high-entropy materials, characterized by their diverse atomic species and non-uniform distributions, the construction and precise control of density functional theory (DFT) models continue to present significant challenges. Future research efforts should concentrate on developing more efficient and accurate computational methodologies through the judicious selection of functionals and basis sets, the application of model preconditioning and simplification strategies, and the utilization of high-performance computing resources. To ensure the fidelity of computational models, it is imperative to establish a model that closely replicates the actual system. This can be achieved by employing structural characterization techniques to validate the consistency between experimental samples and simulation outcomes. For instance, the alignment between experimental and simulated X-ray diffraction (XRD) patterns can provide critical insights into their congruence. Furthermore, a comprehensive analysis incorporating magnetic properties is indispensable, as distinct magnetic orderings (e.g., ferromagnetic, antiferromagnetic) can significantly influence the electronic structure and catalytic behavior of these materials.

### Machine Learning-Accelerated Composition–Property Mapping

By constructing training datasets derived from DFT and complementary sources, and by designing specialized machine learning models, it is possible to accurately screen atomic compositions. This approach can accelerate the prediction of adsorption sites and reaction pathways, thereby enhancing both computational efficiency and accuracy. Ultimately, such strategies are anticipated to substantially reduce the resource requirements of DFT calculations while enabling the targeted design of catalysts for improved performance with respect to specific adsorbates.

### Interdisciplinary Expansion: From Environmental Catalysis to Precision Biomedicine

HELHs demonstrate significant potential not only in catalysis but also in energy conversion, environmental remediation, and biomedical applications. Enhancing interdisciplinary collaborations among chemistry, materials science, computer science, and engineering will offer new momentum for advancing the field and expediting the translation of laboratory research into practical, real-world applications.
